# Revisiting the ecology and evolution of burying beetle behavior (Staphylinidae: Silphinae)

**DOI:** 10.1002/ece3.70175

**Published:** 2024-08-20

**Authors:** Ahva L. Potticary, Mark C. Belk, J. Curtis Creighton, Minobu Ito, Rebecca Kilner, Jan Komdeur, Nick J. Royle, Dustin R. Rubenstein, Matthew Schrader, Sheng‐Feng Shen, Derek S. Sikes, Per T. Smiseth, Rosemary Smith, Sandra Steiger, Stephen T. Trumbo, Allen J. Moore

**Affiliations:** ^1^ Department of Biology Northern Michigan University Marquette Michigan USA; ^2^ Department of Entomology University of Georgia Athens Georgia USA; ^3^ Department of Biology Brigham Young University Provo Utah USA; ^4^ Department of Biological Sciences Purdue University Northwest Hammond Indiana USA; ^5^ Department of Environmental Science Toho University Funabashi Chiba Japan; ^6^ Department of Zoology University of Cambridge Cambridge UK; ^7^ Groningen Institute for Evolutionary Life Sciences University of Groningen Groningen The Netherlands; ^8^ Centre for Ecology and Conservation, Faculty of Environment, Science & the Economy University of Exeter Cornwall UK; ^9^ Department of Ecology, Evolution and Environmental Biology Columbia University New York City New York USA; ^10^ Department of Biology Sewanee, The University of the South Sewanee Tennessee USA; ^11^ Biodiversity Research Center, Academia Sinica Taipei Taiwan; ^12^ University of Alaska Museum and Department of Biology and Wildlife University of Alaska Fairbanks Fairbanks Alaska USA; ^13^ Institute of Ecology and Evolution The University of Edinburgh Edinburgh UK; ^14^ Department of Biological Sciences Idaho State University Pocatello Idaho USA; ^15^ Rocky Mountain Biological Laboratory Crested Butte Colorado USA; ^16^ Department of Evolutionary Animal Ecology University of Bayreuth Bayreuth Germany; ^17^ Department of Ecology and Evolutionary Biology University of Connecticut Waterbury Connecticut USA

**Keywords:** behavioral precursors, life history, Nicrophorini, *Nicrophorus*, parental care ecology

## Abstract

Investigating fundamental processes in biology requires the ability to ground broad questions in species‐specific natural history. This is particularly true in the study of behavior because an organism's experience of the environment will influence the expression of behavior and the opportunity for selection. Here, we provide a review of the natural history and behavior of burying beetles of the genus *Nicrophorus* to provide the groundwork for comparative work that showcases their remarkable behavioral and ecological diversity. Burying beetles have long fascinated scientists because of their well‐developed parenting behavior, exhibiting extended post‐hatching care of offspring that varies extensively within and across taxa. Despite the burgeoning success of burying beetles as a model system for the study of behavioral evolution, there has not been a review of their behavior, ecology, and evolution in over 25 years. To address this gap, we leverage a developing community of researchers who have contributed to a detailed knowledge of burying beetles to highlight the utility of *Nicrophorus* for investigating the causes and consequences of social and behavioral evolution.

## Graphical abstract

Burying beetles of the genus *Nicrophorus* are becoming a model system for the evolution of parental care. Investigating fundamental processes in biology requires the ability to ground broad questions in species‐specific natural history. Here, we leverage a developing community of researchers who have contributed to a detailed knowledge of burying beetles to highlight the ecology and evolution of *Nicrophorus*, and the utility of this genus for investigating the causes and consequences of social and behavioral evolution.

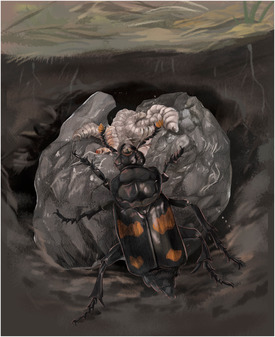

## 1 INTRODUCTION

A major challenge in biology is uncovering general principles through the study of specific organisms (Travis, 2006). It helps to study a diversity of species, but not all species are amenable to detailed studies. One of the advances of recent science is the democratization of “model” species, made possible by technological advances applied to a wide variety of taxa. We now have many more organisms than “the worm” (*Caenorhabditis elegans*), “the fly” (*Drosophila melanogaster*), “the plant” (*Arabidopsis thaliana*), or “the mouse” (*Mus musculus*), that have tractable physiology, ecology, development, genetics, and phylogenetics. This has opened the possibility of addressing broad questions in biology by using biodiversity to understand fundamental biological principles (Travis, 2006). Model organisms develop when a cohesive research community addresses multiple problems at multiple levels within a taxon (Brenner, 2009). Such a model has developed in burying beetles (*Nicrophorus* spp.), which are notable for their extensive parental care. Here, we highlight the utility of this group of insects to address a diversity of biological phenomena, in particular the ecology and evolution of parental care.

The parental behavior of burying beetles (Coleoptera: Staphylinidae: subfamily Silphinae; tribe Nicrophorini; Cai et al., 2022; Sikes et al., 2024) has long fascinated biologists (Fabre, 1918; Milne & Milne, 1944; Pukowski, 1933). *Nicrophorus* show unusually complex parental care for an insect—composed of multiple behaviors including direct interactions with offspring—where parents sequester and prepare carrion nests to provision their larvae (Royle et al., 2012, 2016). Understanding the evolution of parental care is often difficult, as systems that have evolved parental care are often so reliant on care that it is not possible to manipulate care or place it into a comparative framework. For this reason, burying beetles are an excellent model system for the evolution of behaviors like parenting because they are amenable to experimentation and their behavior varies among species; some species raise offspring uniparentally, biparentally, or even communally (Bartlett & Ashworth, 1988; Conley, 1982; Eggert, 1992; Halffter et al., 1983; Müller et al., 2007; Scott & Traniello, 1990; Sun et al., 2014; Wilson & Fudge, 1984). Females and males often show a subset of parental care behaviors when they have a partner but will show the full repertoire of parental care when rearing offspring uniparentally (Cotter & Kilner, 2010; Scott & Traniello, 1990; Smiseth & Moore, 2004). Duration of care differs between individuals, sexes, populations, and species (Scott & Traniello, 1990; Smith et al., 2014; Wilson & Fudge, 1984), and offspring differ in how dependent they are on parental care (facultative versus obligate care; Jarrett et al., 2017, 2018). Beetles of the genus *Ptomascopus* are also of the tribe Nicrophorini and use similar resources yet lack direct offspring feeding and other care behaviors exhibited by *Nicrophorus* (Peck, 1982; Suzuki & Nagano, 2006b; Trumbo et al., 2001). Together, this variation provides a rich experimental and comparative framework to study the causes and consequences of social and behavioral evolution.

All behavior is a response to an organism's experience of a particular context. Thus, an understanding of natural history is central to the investigation of behavior because it allows for tests to be grounded in species‐specific data (Tewksbury et al., 2014). Scott's (1998) review of the ecology, evolution, and behavior of *Nicrophorus* has informed the work of many researchers studying burying beetles. However, there has not been an updated synthesis of *Nicrophorus* ecology and behavior that highlights what we have learned over the past 25 years (Eggert & Müller, 1997; Scott, 1998). Moreover, most research has focused on relatively few North American, European, and Japanese species and the natural history of these taxa has been extrapolated to other members of the genus. Yet, with over 70 extant species of *Nicrophorus* in temperate regions worldwide (Sikes & Venables, 2013), it is clear that “The sanitary officers of the fields are legion” (Fabre, 1918) and most species remain virtually unknown. Here, we extend previous reviews (Eggert & Müller, 1997; Royle & Hopwood, 2017; Scott, 1998) to provide the groundwork for comparative studies that leverage the remarkable behavioral and ecological diversity of *Nicrophorus*, highlighting natural and life‐history data for these species across their life cycle. We then review the current understanding of the evolution of parental care in *Nicrophorus* and suggest some directions for future research.

## 2 BEHAVIORAL ECOLOGY AND LIFE HISTORY

**FIGURE 1:**
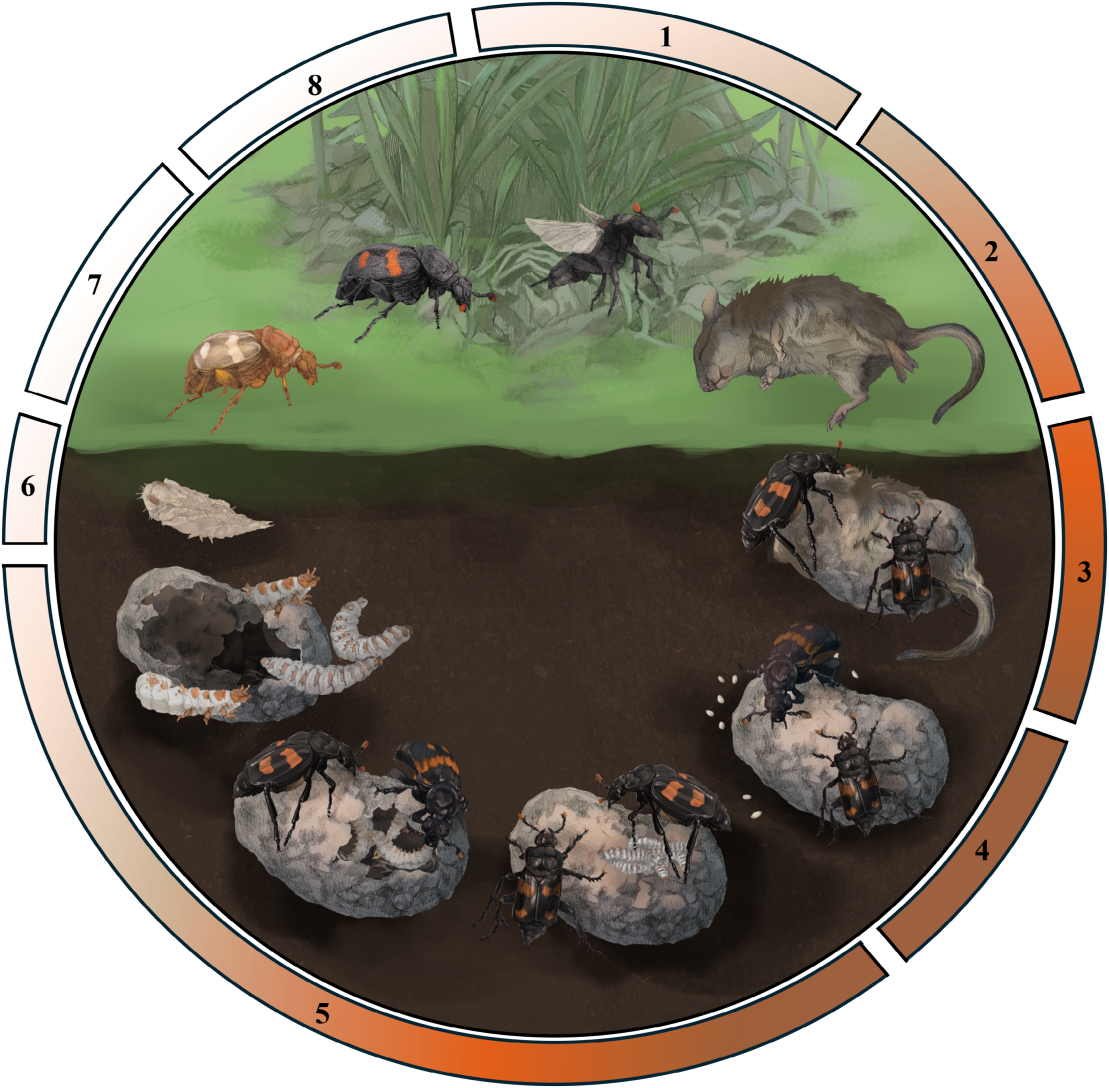
*Nicrophorus* life cycle. (1) Individuals are attracted to carcasses or pheromonally calling males to find a suitable breeding resource, which is (2) often a small vertebrate. (3) Burying beetles work alone or coordinate with one or more individuals to bury the carcass and defend it from potential competitors, parasites, or predators. During burial, adults remove the external covering of the carcass, such as fur, and form the carcass into a brood ball. Parents then cover the carcass with oral and anal secretions—exudate—that regulate microbial communities. (4) Females lay eggs in the soil, and parents spend the embryonic period defending and maintaining the brood ball. Parents eventually cut an incision into the brood ball to create a larval cavity. (5) When the larvae hatch, they crawl to the carcass where parents provision them through oral trophallaxis or larvae self‐feed from the larval cavity. Parents will also regulate brood size through the consumption of some larvae, or filial cannibalism. Larvae remain on the brood ball until dispersing during the third instar stage. (6) Following dispersal, larvae wander to find a location to pupate in the soil. After pupation, they eclose into their adult form (7) and either stay in their underground pupation chamber or emerge from the soil as teneral adults; at this stage, their exoskeleton has not hardened, and they appear light brown in coloration. Eclosed beetles seek food to support sexual maturation. Once their exoskeleton has sclerotized and they have achieved adult coloration, adults become sexually mature within a couple of weeks or longer depending on (8) developmental timing relative to breeding season, overwintering stage, and whether reproductive diapause is needed. Stages 1–2 are nesting resource acquisition, 3–4 are nesting and egg‐laying stages, 5 includes larval stages on the nest, 6–7 post‐parenting offspring development, and 8 is adult natural history. Artwork produced by Kathryn Kollars.

The most notable aspect of burying beetles is their elaborate parental care. Parental care includes multiple behaviors that support the development of offspring, the functions of which differ across the reproductive cycle (Figure 1; Eggert & Müller, 1997; Royle et al., 2016; Scott, 1998). These behaviors can be partitioned into those where parents directly interact with offspring (e.g., offspring provisioning) and those that influence offspring development indirectly (e.g., carcass preparation and maintenance; Duarte et al., 2021; Walling et al., 2008). Below, we briefly discuss the ecology of *Nicrophorus* in five broad categories across the life cycle, including breeding resource acquisition, egg laying and nesting, larval stages on the carcass (nesting resource), post‐parenting offspring development, and adult ecology as it relates to and influences parental care.

### 2.1 Breeding resource acquisition

#### 2.1.1 Finding carcasses

Most burying beetles depend on fresh carrion to breed, an ephemeral resource that is coveted by a wide diversity of vertebrate and invertebrate species (Trumbo, 1992; Wilson & Fudge, 1984). Decomposition rate is not constant but depends on microbial and insect activity, both of which can vary with temperature, season, and habitat (Babcock et al., 2020; Esh & Oxbrough, 2021; Farwig et al., 2014; Ito et al., 2023; Kočárek, 2003; Matuszewski et al., 2010; Müller et al., 2024; Parmenter & MacMahon, 2009; Shean et al., 1993; von Hoermann et al., 2018, 2022). As a result, *Nicrophorus* may have as little as 1–5 days to find a carcass before it becomes unsuitable for breeding (Kočárek, 2003; Smith & Heese, 1995). *Nicrophorus* are thus dependent on strategies that allow them to quickly find and secure fresh carcasses.

*Nicrophorus* are capable fliers (Attisano & Kilner, 2015; Merrick & Smith, 2004). Even species that are very large relative to other *Nicrophorus*, like *N*. *americanus* (Potticary, Belk, et al., 2024), move long distances relative to their body size in search for carrion (Bedick et al., 1999; Creighton & Schnell, 1998; Jurzenski et al., 2011; Raithel et al., 2006). For example, mark–recapture studies of *N*. *americanus* found that individuals searching for carrion traveled 1.23 km a night on average (Creighton & Schnell, 1998) and another study documented an individual that traveled 7.2 km in a single night (Jurzenski et al., 2011). Individuals are thought to find carcasses by detecting the volatile organic compounds that are emitted by microbial metabolism during decomposition (Cernosek et al., 2020; Kalinová et al., 2009; Paczkowski et al., 2012; Shubeck, 1975). Burying beetles sense volatile organic compounds using receptors in the antennomeres of their antennal clubs for long‐distance detection (Boeckh, 1962; Dethier, 1947), and potentially a combination of their antennae and chemosensory centers on their legs at shorter distances (Böhm, 1995; Dethier, 1947; Heinzel & Böhm, 1989; Kalinová et al., 2009). Once a carcass has been found, burying beetles evaluate the suitability of the carcass using a combination of mechanosensory and gustatory cues (Trumbo et al., 1995). Sensory processing appears to vary according to movement strategy, as beetles searching while walking will accept carcasses at different stages of decay or different types of carcasses than free‐flying beetles (Kalinová et al., 2009; Rozen et al., 2008; Smith et al., 2007; Trumbo & Steiger, 2020). Little is known about the movement strategies that enable burying beetles to find carcasses, and how movement strategies influence carcass selection, although some species differ in their preferred flying height while foraging (Ikeda et al., 2011; LeGros & Beresford, 2010; Lowe & Lauff, 2012; Ulyshen et al., 2007; Wettlaufer et al., 2018).

The strength and composition of volatile organic compounds vary across decomposition, resulting in stage‐specific odor bouquets (Recinos‐Aguilar et al., 2019; Trumbo & Newton, 2022; von Hoermann et al., 2013, 2016). *Nicrophorus* and other carrion beetles use variation in the emission of sulfur‐containing volatiles to inform searching behavior (Trumbo & Dicapua, 2021; Trumbo & Steiger, 2020). *Nicrophorus orbicollis* and *N*. *tomentosus* cue in on dimethyl disulfide (DMDS), methyl thiocyanate (MeSCN), and dimethyl trisulfide (DMTS) as simple, long‐distance cues to differentiate stages of carcass decomposition (Trumbo & Steiger, 2020). Small, fresh carcasses emit DMDS and MeSCN, while bloating carcasses and those with higher maggot activity emit more DMDS and DMTS (Armstrong et al., 2016; Chen et al., 2020; Paczkowski et al., 2012; Recinos‐Aguilar et al., 2019; Trumbo & Newton, 2022; Trumbo & Steiger, 2020; von Hoermann et al., 2016). Reproductively active beetles seek fresh carcasses while those seeking food prefer carcasses in bloated or active decay stages (Chapman & Sankey, 1955; Kalinová et al., 2009; Kočárek, 2003; Matuszewski et al., 2010; Peschke & Fuldner, 1987; Reed, 1958; Urbański & Baraniak, 2015; von Hoermann et al., 2013; Wilson & Knollenberg, 1984).

**FIGURE 2:**
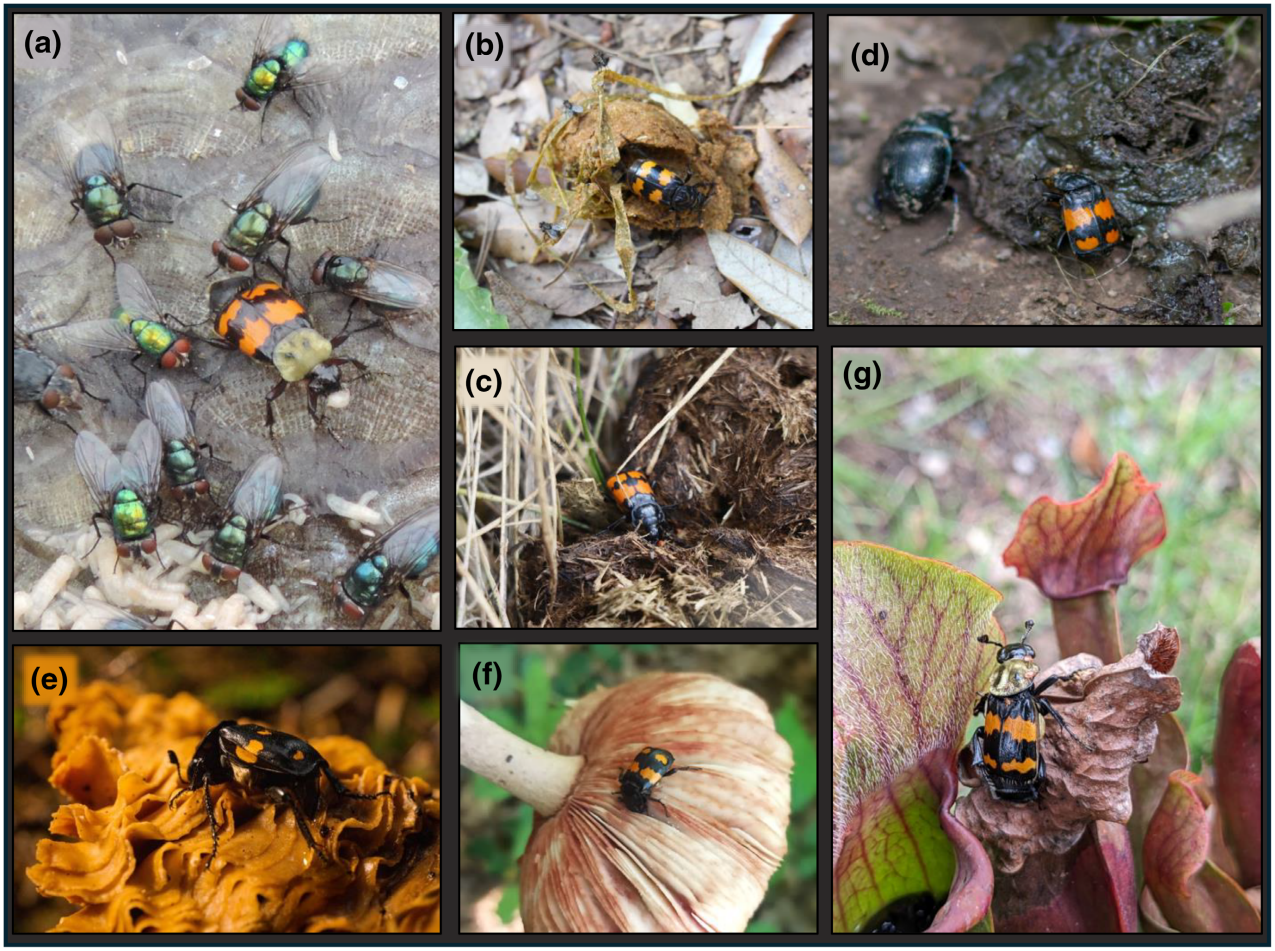
*Nicrophorus* hunt on decomposing carrion, fungi, and dung. Credits for each image are provided in parentheses. *Nicrophorus* eat fly larvae on carrion, as seen by this (a) *N*. *tomentosus* as it consumes a fly larva (Cheri Phillips). In addition, burying beetles forage on both carnivore and herbivore feces, such as this (b) *N*. *interruptus* (Fabien Piednoir), and (c) *Nicrophorus* sp. (Sue Elwell). Some *Nicrophorus* have been observed to hunt adult dung beetles (Pukowski, 1933) like (d) this *Geotrupes* on dung (Alexander Goncharov). *Nicrophorus* also are found on various decomposing fungi, such as *N*. *vespilloides* (e) (Sasha Uhnivenko), and (f) *N*. *defodiens* (Allison Formica). *Nicrophorus* are drawn to a diversity of decomposing substrates, including this (g) potted pitcher plant full of dead insects (Patrick Strzalkowski). Images were collected from iNaturalist observations, without modification, and all permissions for imagery were solicited directly from citizen scientists. Data on observations are provided in Dryad (Potticary, Belk, et al., 2024).

Searching behavior in *Nicrophorus* may be influenced by cues that indicate whether fly larvae are present or absent (Putman, 1978), depending on reproductive state. *Nicrophorus* are not exclusively necrophagous—that is, they do not exclusively eat carrion—and are much better described as necrophilous, or preferring to associate with dead tissue (Fichter, 1949). Adult *Nicrophorus* are known to hunt for invertebrate larvae or adults (Pukowski, 1933), and some *Nicrophorus* species prefer eating fly larvae over carrion when given a choice (Chen et al., 2020; Steele, 1927). A preference for eating invertebrate larvae may explain why *Nicrophorus* are associated with decomposing materials such as dung and fungi, as well as carrion (Balduf, 1935; Clark, 1895; De Jong & Chadwick, 1999; Dekeirsschieter, Verheggen, Lognay, & Haubruge, 2011a; Elton, 1966; Fichter, 1949; Matuszewski et al., 2010; Pukowski, 1933; Steele, 1927). A cursory review of iNaturalist reveals *Nicrophorus* on dung, fungi, compost, eggs, a potted pitcher plant full of dead flies (Figure 2), and in other kinds of microhabitats that include larvae, such as wasp nests (Potticary, Belk, et al., 2024). Artist Abraham Mignon even depicted burying beetles in multiple still‐life paintings of fruit in the mid‐1600s (Mignon 1640–1679a, 1640–1679b). Moreover, *Nicrophorus* can be captured in traps baited with many kinds of carrion (Bedick et al., 2004), and other distinctly non‐carrion substances like vinegar (Nishikawa & Sikes, 2008) and molasses (Katakura & Fukuda, 1975). That *Nicrophorus* are attracted to such a diversity of substances likely reflects that the volatile cues used by *Nicrophorus* to find feeding resources are simple and common to decomposing materials. For example, fungi can also emit DMDS and DMTS (Borg‐Karlson et al., 1994; Lemfack et al., 2014) and are often infested with fly larvae. Hunting larvae is a common behavior to other Silphinae and Staphylinidae; these groups are largely predacious and hunt on decomposing materials (Figure 2d; Young, 2015), as well as in the nests of birds, reptiles, mammals, and insects, such as ants, termites, bees and wasps (reviewed in Voris, 1934). As such, *Nicrophorus* searching behaviors differ across their lifespan based on the likelihood that fly larvae are present, such that breeders prefer materials where flies are absent and feeding adults prefer materials where larvae are likely to be present.

#### 2.1.2 Breeding carcass identity

Little is known about carcass choice in the wild except that breeding *Nicrophorus* typically breed on small, relatively fresh vertebrate carcasses of a preferred size range for their species. Carcass selection behaviors of free‐living *Nicrophorus* are generally cryptic as it is rare and difficult to find carcasses naturally colonized by burying beetles before they are buried. Most experiments place carcasses in the field, using small native mammal and bird species (Lowe & Lauff, 2012; Smith & Merrick, 2001) or commercially sourced mice, rats, and poults (LeGros & Beresford, 2010; Park et al., 2023; Schwindt et al., 2013). Thus, whether individuals or species differ in their carcass preferences under natural conditions is an open question.

There is some evidence suggesting intra‐ and interspecific variation in carcass use. Stable isotope signatures of several sympatric *Nicrophorus* assemblages differed, suggesting differentiation in resource use (Ikeda et al., 2006; Quinby, Feldman, et al., 2020b). *Nicrophorus vespilloides* that bred successfully on mice were more likely to choose to breed on mice in the future (Park et al., 2023), and wild *N*. *vespilloide*s showed seasonal differences in whether individuals were trapped on mice or chicks, though this could reflect differences in age structure across the breeding season (Issar et al., 2024). In addition, the size of the carcass *N*. *vespilloides* develops on, and the amount of care it receives during larval development, influence adult body size and the ability to exploit large carcasses in later life (Schrader et al., 2022). *Nicrophorus investigator* shows no preference between five different rodent species (Smith & Merrick, 2001). However, in other parts of its range, *N*. *investigator* breed on large carrion that are too large to bury such as salmon, and in still other parts of their range, they breed on birds (Hocking et al., 2006, 2007; Peck, 1986; Wilhelm et al., 2001). *Nicrophorus carolina* will use snakes (Arnett, 1946), and *N*. *pustulatus* often breeds on snake eggs (Blouin‐Demers et al., 2004; Blouin‐Demers & Weatherhead, 2000; Smith et al., 2007). Even a single, industrious *N*. *orbicollis* was observed to use a broken open snake egg for reproduction in the laboratory (Smith et al., 2007). Indeed, if it were not for the observation that wild *N*. *pustulatus* do not prepare mice placed on the ground—but will prepare bird carcasses in nests off the ground (DeMarco & Martin, 2020)—their natural history would be assumed to be the same as other *Nicrophorus* based on their behavior in captivity (Smith et al., 2007). Publicly available data such as iNaturalist sightings can improve our understanding of the taxonomic identities of carcasses naturally colonized by various *Nicrophorus* species.

Research has largely focused on the breadth of carcass size that *Nicrophorus* species will use for breeding because carcass size impacts parental egg‐laying and larval strategies (see Egg and Larval stages). The size of a suitable breeding carcass is expected to reflect trade‐offs in a beetle's ability to find, sequester, and prepare it relative to intra‐ or interspecific competitors, producing either species differences in carcass size preference or temporal, habitat, or spatial niche differentiation (Anderson, 1982b; Belk et al., 2021; Eggert & Müller, 1997; Hopwood et al., 2016b; Ohkawara et al., 1998; Royle & Hopwood, 2017; Scott, 1998). For example, *N*. *orbicollis* with larger carcasses are more likely to have conspecific intruders than those with smaller carcasses (Trumbo, 1991) and broods fail on carcasses that are not of the appropriate size (Smith & Heese, 1995). The preference for fresh carcasses may reflect the diminishing nutritional value of carcasses as they decompose and greater consumption of the carcass by other insects like flies (Kočárek, 2003). Thus, securing a fresh carcass improves nutrient availability and quality for larvae across developmental stages (Rozen et al., 2008; Trumbo, 2016) and reduces the threat of predation to young (see Larval stages).

### 2.2 Securing the carcass: competition, burial, and preparation

#### 2.2.1 Competitive behavior

Competition for breeding carcasses is likely a major driver in the evolution of parental care in burying beetles. Intra‐ and interspecific competitors reduce the availability of breeding carcasses through exploitation—by reducing the quantity or quality of carcasses—or by interference competition—by hindering access to the carcass through fighting or behaviors that decrease the salience of cues that may allow other individuals to find the carcass. Both intraspecific and interspecific competition are important in the burying beetle system. For example, the range limits of *N*. *nepalensis* are defined by exploitative competition with blowflies at their cool, upper elevation boundary, and by interference competition with blowflies at the warm, lower elevation boundary (Chan et al., 2019). Larger individuals typically win in direct contests among burying beetles over carcasses (Bartlett & Ashworth, 1988; Lee et al., 2013; Otronen, 1988; Pukowski, 1933; Scott, 1994a; Smith & Belk, 2018a; Steiger et al., 2012), although nutritional status also influences contest outcomes (Hopwood et al., 2014). The role of body size in fights has been well described (Eggert & Müller, 1997; Royle & Hopwood, 2017; Scott, 1998).

However, body size is relative and thus the importance of body size depends on the competitive environment that a beetle encounters, such as the size and relative condition of other burying beetles in the population (Creighton, 2005; Hopwood et al., 2014, 2016b). For *Nicrophorus* studied thus far, both sexes will fight for breeding carcasses, but the outcome of contests based on body size can differ between sexes (Smith & Belk, 2018a). Whether a contest will occur depends on the sex and size of the beetle that finds the carcass. For example, previously mated females can immediately bury a carcass, reducing the likelihood of a contest, whereas a male that discovers a carcass often emits pheromones to call for females, increasing opportunities for a fight as other beetles are drawn to the carcass (Haberer et al., 2008; Hopwood et al., 2016a; Royle & Hopwood, 2017).

Intraspecific fights can result in injuries such as lost legs and antennae, and even death (Komdeur et al., 2013; Scott & Traniello, 1990; Trumbo, 1990c, 1992, 2007). Fighting behaviors include pushing, biting, flipping an opponent over, and in the most intense cases, mounting/grappling that superficially resembles copulatory behavior, and death by dismemberment (Otronen, 1988; Suzuki, 2000). The nicrophorine *Ptomascopus morio* also fights near carcasses, including pushing, biting, and male–male mounting, although only males compete in this species (Suzuki et al., 2005; Suzuki & Nagano, 2006b). Body size varies extensively within all *Nicrophorus* studied to date (Otronen, 1988), and thus, it is likely that beetles often employ alternative seeking or sequestering strategies that do not depend on combat (Ohkawara et al., 1998) because competition may also include non‐aggressive behaviors like finding and burying carcasses quickly.

#### 2.2.2 Burial

Why do burying beetles bury carcasses? Burial serves multiple purposes, such as protecting the brood ball from intruders, regulating temperature, and as a defense against desiccation. Early colonizing *Nicrophorus* can secure a carcass by finding it quickly, moving and burying it, and reducing the salience of the microbial cues that allowed them to find the carcass (Milne & Milne, 1944, 1976; Trumbo, 2023). These activities are types of interference competition that do not require fighting and thus are suitable strategies for beetles of all sizes to secure a breeding resource (Bartlett & Ashworth, 1988; Duarte et al., 2018; Shubeck & Blank, 1982; Shukla, Vogel, et al., 2018b; Smith, Bonilla, et al., 2000a; Smith & Merrick, 2001; Trumbo & Sikes, 2021), although beetles of different sizes may differ in how deep they can bury (Eggert & Sakaluk, 2000). While burial greatly reduces the likelihood of discovery of the brood ball by intruders, it does not preclude it (Hopwood et al., 2015; Payne et al., 1968; Rodriguez & Bass, 1985; Shubeck & Blank, 1982; Trumbo & Sikes, 2021), and consequently, intrusions, takeovers, and brood parasitism are common in some species (Niida et al., 2024; Scott, 1994b; Suzuki, 2000, 2004, 2006a; Trumbo, 1990c, 1994). Burial depth (Potticary, Belk, et al., 2024) affects whether intruders can find brood balls (Shubeck, 1985; Shubeck & Blank, 1982), with fewer intrusions by other beetles, flies, and vertebrate scavengers the deeper a carcass is buried (Rodriguez & Bass, 1985; Scott & Traniello, 1990; Trumbo, 1990c; Wilson, 1983; Wilson & Knollenberg, 1987; Zou et al., 2022).

Moreover, burial creates a crypt with a less variable temperature than at the soil surface (Rodriguez & Bass, 1985). Growth rates, adult body size, and fecundity are all influenced by temperature (Angilletta et al., 2004; Atkinson, 1994; Kingsolver & Huey, 2008; Meierhofer et al., 1999). Higher temperatures increase egg mortality in *N*. *quadripunctatus* (Nisimura et al., 2002). *Nicrophorus orbicollis*, for instance, bury carcasses deeper when ambient surface temperatures are higher, and deeper crypts have dampened temperature fluctuations (Harrison, 2021). Average larval mass decreases as the temperatures become more variable in *N*. *orbicollis* (Harrison, 2021), at warmer temperatures for *N*. *marginatus* (Keller et al., 2021), and at lower elevations in *N*. *investigator* (Smith, Hines, et al., 2000b). Even short heat waves impact parenting and offspring development in *N*. *vespilloides* (Pilakouta et al., 2023). Finally, microhabitat selection influences larval development; *N*. *investigator* parents prefer to bury carcasses in sunny alpine meadows where larvae develop faster than in shaded forests, even though adults forage in both habitat types (Smith & Heese, 1995).

Temperature and competition as drivers of burying behavior are not mutually exclusive. Ambient temperature variation influences the likelihood and diversity of intruders by altering the release of volatiles from the carcass (Potticary, Otto, et al., 2023b; Shean et al., 1993; Wilson et al., 1984). Temperature can also change the nature of competitive interactions between burying beetles and other species at the carcass, such as phoretic mites and blowflies (Chen et al., 2020; Sun & Kilner, 2020), and perhaps even microbes and burying beetle larvae (Grew et al., 2019). Given the hypothesized importance of reducing opportunities for competition at the brood ball, it is unclear why burial depth varies so much within and across *Nicrophorus*; ranging from shallow crypts that do not cover the carcass, to deep burials several centimeters below the surface, to no burial at all (e.g., when the carcass is too large to bury). This variation may reflect a trade‐off between environmental context (e.g., burial substrate and abundance of competitors), physiology, and the energetic costs of burial (Wilson, 1983; Wilson & Knollenberg, 1987). For example, smaller species may not be able to bury carcasses as deep as larger species or are unable to sequester a larger carcass when competition is high, although this is a hypothesis that requires testing.

#### 2.2.3 Preparation of the brood ball: Shaving, rounding, incisions, and exudate

Throughout the burial process, *Nicrophorus* parents perform a complex series of activities to prepare the carcass, including rounding, removing the external covering (e.g., fur or feathers), and coating the carcass in exudate secretions, and cutting an incision in the carcass (Duarte et al., 2018; Hwang & Lin, 2013; Pukowski, 1933; Wang & Rozen, 2017). Similar to negotiations between parents at larval stages (see Larval stages), male *N*. *orbicollis* modulate their carcass preparation activity in response to female activity (Creighton et al., 2015). These activities are expected to mitigate the costs of developing on carrion, a microbe‐rich and potentially putrefying resource much coveted by other organisms (reviewed by Körner et al., 2023). Carcass preparation can reduce the discovery of the brood ball by other insects (Chen et al., 2020; Trumbo et al., 2021; Trumbo & Sikes, 2021) and can support offspring development by managing the microbial community (Shukla, Plata, et al., 2018a; Trumbo, 2017).

**FIGURE 3:**
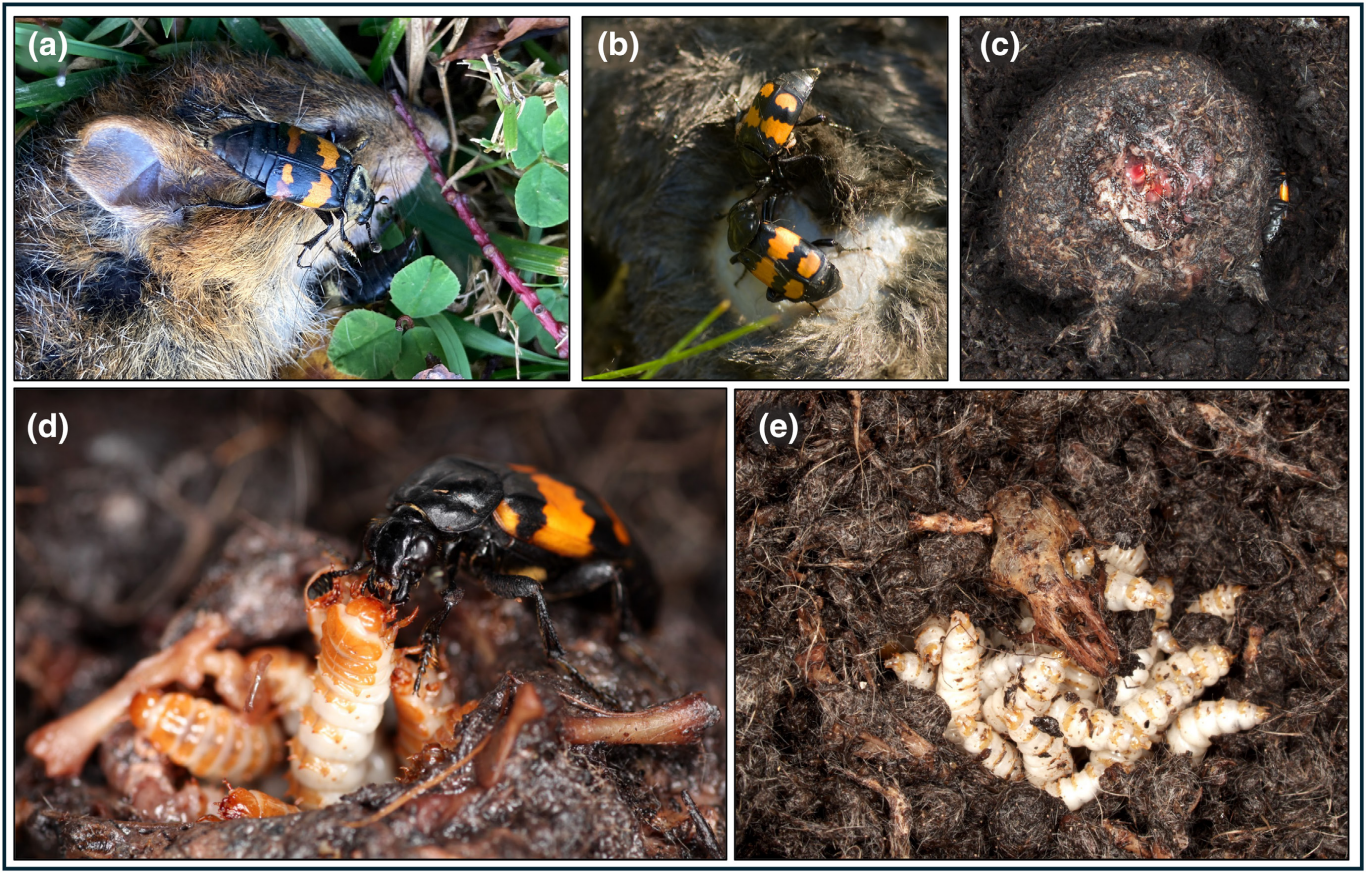
Parenting of *Nicrophorus*. Credits for each image are provided in parentheses. Reproductive activity is initiated when a *Nicrophorus* finds a suitable breeding carcass, like this (a) *N*. *tomentosus* on a mouse (Diane Pfeiffer). Parents then remove the external covering of the carcass, like these (b) *N*. *vespilloides* shaving a small mammal (Thierry Arbault). The carcass is then (c) rounded, and a small incision is made in the skin of the brood ball (Paul Hopwood). Once larvae hatch, parents (d) like this *N*. *vespilloides* will regurgitate carrion to their offspring (Nick Royle). Larvae are fed by parents and/or self‐feed from the brood ball until (e) it has been completely consumed in the third larval instar, when larvae disperse (Paul Hopwood). Images collected from iNaturalist observations were not modified and all permissions for imagery were solicited directly from citizen scientists. Data on observations are provided in Dryad (Potticary, Belk, et al., 2024).

How do carcass preparation behaviors accomplish these tasks? Parents remove the external coverings of the carcass and round the carcass, resulting in a clean meatball with the skin intact except for an incision cut on top in which *Nicrophorus* larvae congregate (Figure 3). Parents often do not shape large carcasses into a ball. However, even an *N*. *investigator* breeding on a rabbit carcass cleaned and maintained the area around their brood (Peck, 1986). Such “shaving” behavior may serve multiple purposes, including reducing volatile cues and fly eggs, as well as facilitating the application of exudate that slows decomposition. Decomposing proteins, like the keratin of fur and feathers, can produce DMDS and DMTS as byproducts (Dekeirsschieter et al., 2009) and removing these structures could reduce the emission of volatiles that are attractive to other necrophilous organisms (Woodard, 2006). However, removed fur and other external coverings are often present, lining the crypt, so it is unclear how this would reduce volatile emission. Moreover, fly eggs are often laid on the external coverings of carcasses, such as fur or feathers. Wilson (1983) never observed several North American burying beetle species specifically hunt or destroy fly eggs, and hypothesized that removing the external covering was an indirect mechanism to reduce dipteran infestation.

Carcass rounding could be an artifact of how burying beetles bury carcasses and develop the crypt (Pukowski, 1933), yet burying beetles sometimes round carcasses even in the absence of burial or a full crypt. A rounder carcass may lower the cost of producing antimicrobial exudates for parents by reducing the area to be covered (Duarte et al., 2021). Moreover, rounder *N*. *vespilloides* brood balls are less hospitable to blowfly larvae (Sun & Kilner, 2020). However, carcass roundness did not affect offspring outcomes like brood size of *N*. *vespilloides* (Duarte et al., 2021), and *N*. *orbicollis*, *N*. *pustulatus*, and *N*. *vespilloides* larvae can survive on unprepared carcasses in a laboratory (Capodeanu‐Nägler et al., 2016; Trumbo, 2017). Carcass rounding could reduce cues to potential intruders, as areas of greater volatile release (e.g., the mouth of the carcass) and any incisions in the carcass are either placed in the interior of the brood ball or are actively covered by parents (Trumbo, 2017).

There may be a trade‐off between the creation of the larval cavity to support larval growth and the release of microbial compounds that attract other insects (Trumbo, 2017), as incisions in the skin of a carcass increase the rate of decomposition and release of volatiles (Brodie et al., 2014; Ito et al., 2023; Recinos‐Aguilar et al., 2019). *Nicrophorus vespilloides* larvae depend on an incision in the carcass to self‐feed (Duarte et al., 2021; Eggert et al., 1998) and *N*. *pustulatus* will cut an incision in otherwise unprepared snake eggs for their larvae (Smith et al., 2007). In the absence of parents, *N*. *vespilloides* larvae are much more successful with an incision (Eggert et al., 1998; Jarrett et al., 2018). Parents mediate this trade‐off by changing the timing of when they place the incision; only 26% of *N*. *vespilloides* parents place an incision in the carcass before larvae hatch (Duarte et al., 2021), and *N*. *orbicollis* parents will patch any incisions that occur before hatching (Trumbo, 2017). Together, these data support the idea that rounding and shaving reduce the potential for intruders at the carcass, rather than directly benefiting offspring development (Capodeanu‐Nägler et al., 2016; De Gasperin et al., 2016; Duarte et al., 2021), while incisions in the carcass support larval self‐feeding and aggregation (Duarte et al., 2021; Smith et al., 2007). Tests of these hypotheses are needed in the wild.

Carcasses are rapidly colonized by bacteria on the surface and burial itself increases microbial diversity (Duarte et al., 2018). High microbial loads on older carcasses can negatively affect offspring development and the absence of carcass preparation can lead to total brood failure (Arce et al., 2012; McLean et al., 2014; Rozen et al., 2008; Trumbo, 2016), but larvae can survive without any carcass preparation in some situations (Capodeanu‐Nägler et al., 2016, Trumbo, 2017). *Nicrophorus* actively mitigate microbial challenges on the carcass that offspring would otherwise experience (Körner et al., 2023). When *N*. *vespilloides* discovers a carcass, parents alter the composition and function of their anal secretions, or exudate, from defense‐only in non‐breeding individuals to a parental function in breeders (Arce et al., 2012; Cotter & Kilner, 2010; Palmer et al., 2016; Steiger et al., 2011). Exudate production is considered costly because it trades off fecundity and aspects of personal immunity (Cotter et al., 2010, 2013; Cotter & Kilner, 2010; Reavey et al., 2014). *Nicrophorus vespilloides* parents alter the antibacterial activity of their exudate when another parent is present, also suggesting that exudate is costly (Cotter & Kilner, 2010).

The exudates of both parents and larvae contain antimicrobial compounds, including lysozymes, antimicrobial peptides, and microbes that can inhibit some bacteria and fungi (Arce et al., 2012, 2013; Cotter & Kilner, 2010; Duarte et al., 2018; Hall et al., 2011; Hwang & Lin, 2013; Jacobs et al., 2016; Palmer et al., 2016; Parker et al., 2015; Steiger et al., 2011; Suzuki, 2001; Vogel et al., 2017). When placed on the skin of the brood ball, these secretions can act as a defense against microbial challenge and decomposition (Arce et al., 2012; Hoback et al., 2004; Rozen et al., 2008; Vogel et al., 2017), and reduce volatile cues other organisms use to locate carcasses (Duarte et al., 2018, 2021; Shukla, Plata, et al., 2018a; Trumbo et al., 2021; Trumbo & Sikes, 2021; Trumbo & Steiger, 2020). Parents mediate the microbial community by weeding—that is, reducing the abundance of some microbes—and seeding, or increasing the abundance of other microbes relative to unprepared carcasses (Duarte et al., 2018; Miller et al., 2019). Both methods of mediating the microbial community can support larval development. For example, parental seeding of *Yarrowia* species can produce a biofilm‐like matrix that supports larval growth by forming an interface between larvae and the brood ball (Shukla, Plata, et al., 2018a; Vogel et al., 2017). Larvae acquire the microbiota and antimicrobial peptides of their parents from the brood ball and parental regurgitations to larvae, which serve as inocula for larval digestive tracts (Miller et al., 2019; Shukla, Vogel, et al., 2018b; Vogel et al., 2017; Wang & Rozen, 2017; Ziadie et al., 2019).

Microbial communities may reasonably be expected to differ across habitats and substrates, and species differ in how carcasses are prepared which could affect how well offspring acquire nutrients from the brood ball. For example, *N*. *vespilloides* that usurped *N*. *quadripunctatus* carcasses were not as successful as individuals raising larvae on a carcass they prepared themselves (Suzuki, 2004). Furthermore, the antimicrobial exudate compounds of *N*. *marginatus* and *N*. *carolina* differed—even though they were captured in the same habitat (Woodard, 2006)—depending on temperature and food source (Jacques et al., 2009), perhaps reflecting differences in resource use. However, how parents mitigate microbial variation across ecological contexts to support offspring development has not been examined. There is some evidence from laboratory studies that parents alter exudate production in response to variable social and ecological conditions. For example, female *N*. *vespilloides* increase the antibacterial activity of their exudate in response to bacterial challenge on the brood ball (Cotter et al., 2010), and decrease antibacterial activity when phoretic mites are present (Duarte et al., 2017). Future research may investigate how variation in microbial environment influences parental behavior and how parents mitigate the costs of their offspring growing up on carrion, such as by mitigating microbial challenge (Körner et al., 2023).

### 2.3 Mating, oviposition, and eggs

**FIGURE 4:**
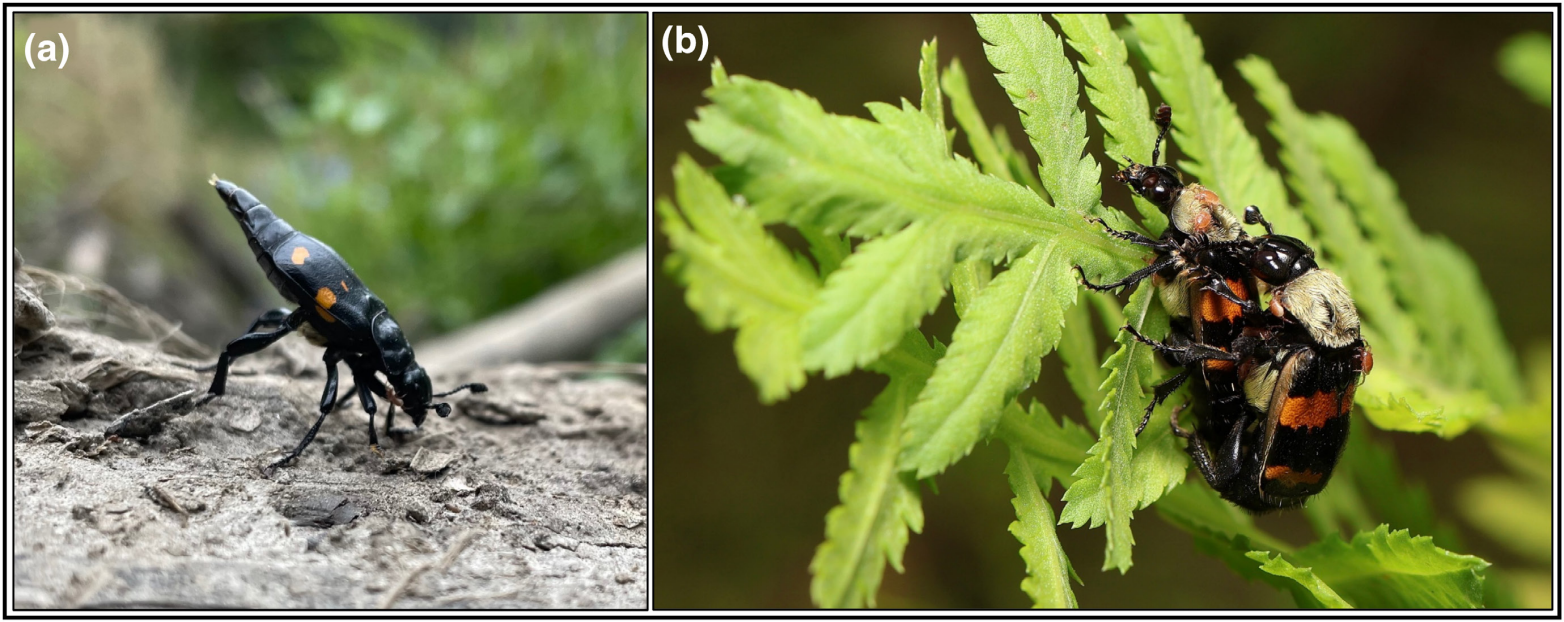
Calling and mating in *Nicrophorus*. Credits for each image are provided in parentheses. (a) Males like this *N*. *defodiens* may call for females by releasing pheromones in a distinctive, abdomen‐up position (Matthias Morse). Mating then occurs either on a breeding carcass or (b) in another location, like these *N*. *tomentosus* copulating on a fern (Mauro Brum). Images collected from iNaturalist observations were not modified and all permissions for imagery were solicited directly from citizen scientists. Data on observations are provided in Dryad (Potticary, Belk, et al., 2024).

Mating in *Nicrophorus* occurs at carcasses (Sakaluk & Müller, 2008) or females may be attracted to males emitting pheromones on or off a carcass (Figure 4; Beeler et al., 1999; Chemnitz et al., 2015; Eggert, 1992; Eggert & Müller, 1989a, 1989b; Müller & Eggert, 1987; Smith et al., 2007). Females that appear at carcasses often carry viable sperm (Müller & Eggert, 1989), though sperm stored in the spermatheca of *N*. *vespilloides* start to become infertile 3 weeks after insemination (Eggert, 1992). For cases where males are present when females are laying eggs, mating is frequent during egg laying but ceases when larvae arrive (Engel et al., 2014). Juvenile hormone (JH) in females of *N*. *vespilloides* and *N*. *orbicollis* increases from carcass discovery until larvae hatch (Engel et al., 2016; Trumbo, 1997), triggering the production of an anti‐aphrodisiac that reduces male mating behavior during larval stages (Engel et al., 2016, 2019).

Even females with viable sperm stored do not mature eggs until they have found and assessed a suitable breeding carcass (Huerta, 1991; Scott & Traniello, 1987; Trumbo et al., 1995; Wilson & Knollenberg, 1984). Female condition and assessment of the carcass influence the number of eggs that females lay, and the eventual regulation of brood size, which together serve to regulate the amount of carrion available to each offspring (Bartlett & Ashworth, 1988; Creighton, 2005; Müller et al., 1990; Nagano & Suzuki, 2007; Scott, 1997; Smith et al., 2015; Steiger, Richter, et al., 2007b; Trumbo & Fernandez, 1995; Wilson, 1983). *Nicrophorus pustulatus* produces the largest clutches observed in *Nicrophorus*—which can be upwards of 150 eggs versus the usual range of 20–75—which may reflect an adaptation to using clusters of reptile eggs for reproduction (Blouin‐Demers & Weatherhead, 2000; Smith et al., 2007; Trumbo, 1992). The number of eggs laid can greatly exceed the number of larvae that eventually leave the carcass (Bartlett, 1987), and while filial cannibalism of larvae can regulate larval numbers, it is also possible that parents lay additional eggs because not all eggs survive to hatch; for example, due to predation by phoretic mites (Beninger & Peck, 1992; Wilson, 1983). Indeed, when parents of *N*. *vespilloides* were inhibited from committing filial cannibalism of larvae in experimental evolution, egg hatchability increased, suggesting that parents typically produce extra infertile eggs (Rebar et al., 2022). Parents may produce extra, infertile eggs to reduce the likelihood of fertile eggs being eaten. Other factors like social environment influence egg laying; for example, *N*. *vespilloides* females lay more, and larger, eggs when breeding jointly with another female than when breeding on their own (Eggert et al., 2008; Richardson & Smiseth, 2020). How variation in egg laying relates to ecological pressures like predation, phoretic mite load, desiccation, or other threats is unknown.

Egg laying can occur over 30 h in *N*. *vespilloides*, leading to asynchronous hatching (Smiseth et al., 2008), although the last laid eggs develop slightly faster (Smiseth, Ward, & Moore, 2006b). Embryonic developmental rates are influenced by egg size and temperature though few data are available on the embryonic periods of *Nicrophorus* (Table 1), or how ecological conditions alter aspects of embryonic development. Species vary in where they lay eggs relative to the carcass (Anduaga, 2009; Pukowski, 1933), so it is possible, but not yet shown, that females can influence embryonic development based on egg placement.

**TABLE 1:** Life‐history transitions.

Species	Embryonic period	Hatch to dispersal	Dispersal to eclosion
*N*. *concolor*	3–4	8–9	29–34
*N*. *defodiens*	2–4	6–7	25–37
*N*. *guttula*	4–7	7–14	29–40
*N*. *investigator*	5–6	10–11	Overwinter
*N*. *marginatus*	3–5	7–14	30–40
*N*. *mexicanus*	2–5	10–13	37
*N*. *nepalensis*	2–3	11–13	30–45
*N*. *nigrita*		~12	~68
*N*. *orbicollis*	4	7–9	30
*N*. *pustulatus*	3–4	7–9	28–30
*N*. *quadripunctatus*	3	6	37
*N*. *sayi*	8	13–14	55
*N*. *vespilloides*	2–3	5–7	14–20
*Ptomascopus morio*	4–7	10	58–70

*Note*: *Nicrophorus* species and close relative *Ptomascopus morio* show wide variation in the length of developmental periods. These data were collected in laboratories that differ in their rearing temperatures, which is expected to influence life‐history transitions. These data are primarily from species that overwinter as adults rather than other stages (e.g., prepupae). Developmental periods are presented as the number of days of the embryonic period (egg laying to hatch), hatch to dispersal from the carcass (first, second, and part of third instar), and dispersal to eclosion (conclusion of third instar and pupal period). The pupal period is not parsed out because the length of this stage is rarely reported. The period from eclosion to sexual maturity is rarely reported and is unknown for most taxa. References are provided in Appendix S1.

### 2.4 Larval stages on the brood ball

Once larvae hatch, they crawl to the carcass and group in the larval cavity made by parents. How larvae find their nest is unknown, especially in instances where carcasses are large and multiple groups are breeding (Hocking et al., 2006). Larvae can be drawn to parental stridulations, produced by rubbing elytra on the abdomen (Niemitz & Krampe, 1972). However, larvae can also locate a brood ball when parents are absent. In this case, larvae are perhaps also drawn to the cues of decay emitted from the incisions that parents create (Smith et al., 2007; Trumbo, 2017). *Nicrophorus* parents cannot recognize their larvae and instead employ temporal cues, where any larvae arriving on the carcass at the time that the parents expect their offspring are accepted and fed, whereas those that appear too early are eaten (Benowitz et al., 2015; Eggert & Müller, 2000; Komdeur et al., 2013; Müller & Eggert, 1990; Oldekop et al., 2007; Potticary, McKinney, et al., 2023a). This lack of offspring recognition can be taken advantage of by intra‐ and interspecific brood parasites (Eggert & Müller, 2000; Müller & Eggert, 1990; Scott, 1997; Smith & Belk, 2018b; Trumbo, 1994). The cue that “starts the clock” for temporal recognition is egg laying for females (Oldekop et al., 2007) and probably the frequency of copulation for males, since these activities coincide and both parents begin to accept larvae at roughly the same time in *N*. *vespilloides* and *N*. *orbicollis* (Oldekop et al., 2007; Potticary, McKinney, et al., 2023a).

The brood ball is the only source of nutrition for developing young until they have eclosed into their adult form. Consequently, carcass size relative to brood size is interconnected with brood mass and beetles that receive poor nutrition or poor parental provisioning during larval stages are smaller as adults (Bartlett, 1987; Bartlett & Ashworth, 1988; Jarrett et al., 2017; Kozol et al., 1988; Lock, 2012; Potticary, Cunningham, et al., 2024; Schrader et al., 2018; Scott & Traniello, 1990; Sikes, 1996; Smiseth et al., 2014; Smith, 2002; Trumbo & Xhihani, 2015; Wilson & Fudge, 1984), a pattern also observed in other non‐parental silphines (Ratcliffe, 1972). Parents commit infanticide to alter offspring number relative to carcass volume (Bartlett, 1987; Smith & Belk, 2018b; Trumbo, 1990b; Trumbo & Fernandez, 1995). Such filial cannibalism may be released by offspring begging; in *N*. *vespilloides*, begging larvae were more likely to be eaten by parents (Andrews & Smiseth, 2013), perhaps indicating that the degree of offspring begging is an indirect cue to parents that there are too many larvae relative to carcass volume. Filial cannibalism mediates larval competition by influencing the trade‐off between the number, size, and survival offspring (Bartlett & Ashworth, 1988; Creighton, 2005; Creighton et al., 2009; Potticary, Cunningham, et al., 2024; Scott & Traniello, 1990; Trumbo, 1990b; Wilson & Fudge, 1984), though the sexes can differ in how effectively they mediate this trade‐off. For example, male *N*. *orbicollis* cull proportionally more larvae across carcass sizes than females, while females are better able to match brood size to carcass size (Smith et al., 2015). Parents also mediate brood size relative to ecological conditions; for example, *N*. *orbicollis* adjust brood number and offspring body size in anticipation of how competitive the environment may be, such as due to burying beetle density (Creighton, 2005).

**FIGURE 5:**
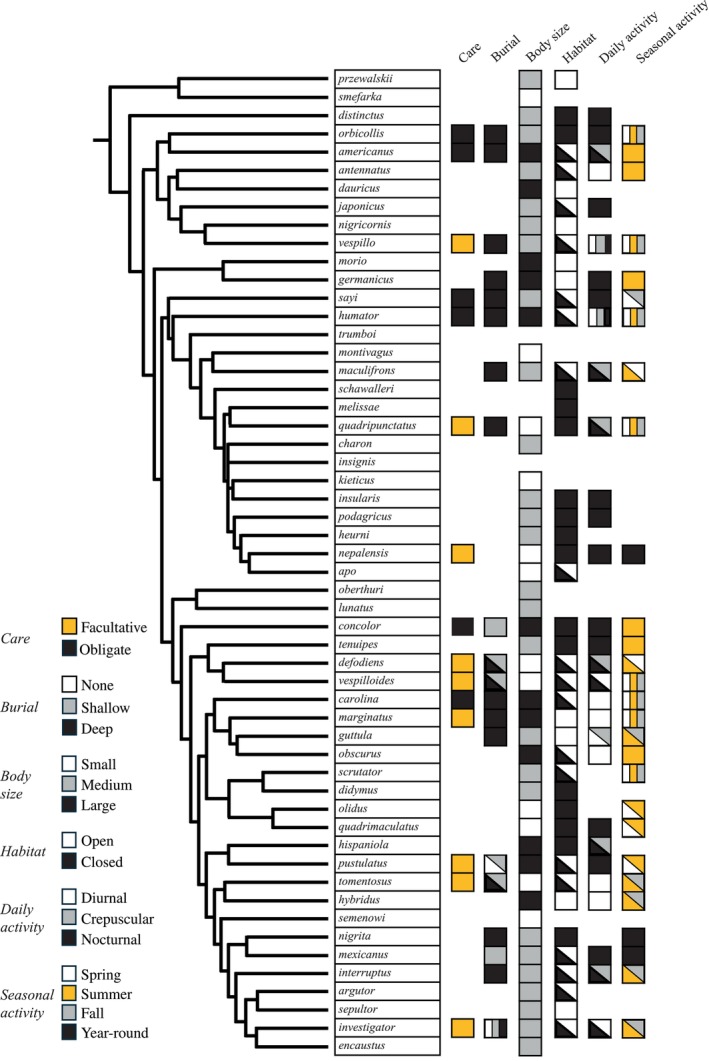
Natural history of *Nicrophorus*. Partial phylogeny of subfamily *Nicrophorus* modified from Sikes and Venables (2013). Key to fields in each column of data is provided on the left. Missing squares indicate species for which data are unknown. Care is the dependency of offspring on post‐hatching parental care, with species that require post‐hatching care (*obligate*) and do not require post‐hatching care (*facultative*). Burial refers to the burial depth of carcasses during breeding, including burials below leaf litter or <2 cm below the soil surface (*shallow*), >2 cm below the soil surface (*deep*), or not buried at all (*none*). These categories only include burials where the beetles inter the carcass from the soil surface, excluding those that were dragged into burrows. Body size was determined by parsing data from all species worldwide into three separate categories based on quartiles, 1%–25% (*small*), 26%–75% (*medium*), and 75%–100% (*large*). Only studies where body size was measured using pronotum width as a proxy were included. Given the breadth of habitat types occupied by *Nicrophorus* worldwide, Habitat data were defined as *open* or *closed*. Open habitats are those with minimal or absent canopy cover (e.g., steppe and meadows), while closed habitats are those that have moderate to extensive canopy (e.g., forests). Data reflect locations where *Nicrophorus* have been documented but are not necessarily exclusive associations, particularly since *Nicrophorus* seeking food may use a broader range of habitats than beetles will breed in. Daily activity includes species that are diurnal, nocturnal, or crepuscular. Seasonal activity includes species that are active in the spring, summer, fall, or year‐round. As seasonal activity differs depending on the location of sampling, these data represent seasonal associations observed for each taxon across its range, which obscures local variation in seasonality. References are provided in Appendix S1. Body size and iNaturalist data are included in Dryad (Potticary, Belk, et al., 2024).

Larval development depends on how efficiently the developing beetle acquires nutrients, involving a combination of *direct care*—the social interaction where parents regurgitate to offspring—and *indirect care*—parental processing and maintenance of the brood ball (Walling et al., 2008). In some species, larvae require at least some post‐hatching care (obligate care), whereas in other species, larvae do not require parents because they are equipped to self‐feed upon hatching (Milne & Milne, 1976, facultative care, Figure 5; Capodeanu‐Nägler et al., 2016; Jarrett et al., 2017). The parenting that larvae receive changes not only whole‐body developmental metrics like offspring growth rate and body size but also induces changes in the timing of development of larval systems on ecological and evolutionary timescales (Attisano & Kilner, 2015; Benowitz, Amukamara, et al., 2019a; Meierhofer et al., 1999; Rauter & Moore, 2002). For example, *N*. *vespilloides* larvae downregulate genes associated with immune defenses when parents are present, potentially as a response to social immunity conferred by parents (Ziadie et al., 2019). Variation in the family environment can alter the timing of larval behavior; parents with larger broods provide less direct care to individual larvae, and as a result, larvae in large broods switch to predominantly self‐feeding earlier than larvae in small broods (Smiseth et al., 2007). Parenting can produce changes in developmental timing over evolutionary time as well. For example, serrations on larval mandibles are thought to facilitate self‐feeding and these are present at hatching for multiple facultative care species, but missing until the second instar in obligate care species (Benowitz et al., 2018). *Nicrophorus* larvae are less sclerotized in early instars than *Ptomascopus* and far less sclerotized than Silphini larvae (Anderson, 1982a), which likely results from a decreased need to invest in larval traits involved in predator defense due to parental guarding.

The outcome of social interactions between parents and offspring depends on the degree of correspondence between their physiology and behavior. At minimum, the nature of this interaction differs based on variation in larval begging (Bladon et al., 2023; Capodeanu‐Nägler, Eggert, et al., 2018a; Smiseth et al., 2003, 2007; Smiseth & Moore, 2008), offspring dependency on parent feeding (Capodeanu‐Nägler et al., 2016, Jarrett et al., 2017), timing and frequency of parental regurgitations (Benowitz et al., 2016; Smiseth et al., 2007), duration of post‐hatching care (Bladon et al., 2023; Scott & Traniello, 1990), the number, sex, age, size, and condition of parents present (Bartlett & Ashworth, 1988; Benowitz et al., 2013; Jarrett et al., 2018; Lambert & Smiseth, 2024; Scott & Traniello, 1990; Smith et al., 2014; Steiger, 2013), and the time of year (Scott & Traniello, 1990). There are strong indirect genetic effects and covariances between parents and offspring coordinating their social interactions (Head et al., 2012; Lock et al., 2007; Parker et al., 2015; Walling et al., 2008).

That parental care is an interacting phenotype between parents and offspring is well illustrated by the result that in some species, larvae are larger when raised by their parents, while in others, larvae are larger when raised by another species (Benowitz et al., 2015; Capodeanu‐Nägler, Ruiz de la Torre, et al., 2018b; Jacques et al., 2009; Smith & Belk, 2018b). While this pattern could reflect species differences in the number of offspring raised or social interactions between parents and larvae, parents within and across species may prepare the carcass differently or exude different compounds, perhaps altering offspring development independently of direct care (Bladon et al., 2023; Duarte et al., 2021; Suzuki, 2004; Woodard, 2006). Lastly, developmental rates differ between taxa, independently of the care received (Benowitz et al., 2015). Parental care and life history are intricately connected (Belk et al., 2021; Billman et al., 2014; Creighton et al., 2009; Wang et al., 2021), which makes the lack of life‐history transition data for most *Nicrophorus* species unfortunate (Table 1). It would be a valuable avenue for future research to determine the reciprocal interaction between parental care and life‐history evolution in other species of Silphinae.

Burying beetles are usually described as having biparental care (Milne & Milne, 1976), but this is an oversimplification as within a species parents can successfully raise offspring uniparentally (either by a single female or male), biparentally, or even communally (Bartlett & Ashworth, 1988; Benowitz & Moore, 2016; Conley, 1982; Eggert, 1992; Halffter et al., 1983; Komdeur et al., 2013; Ma et al., 2022; Müller et al., 2007; Pukowski, 1933; Scott & Traniello, 1990; Tsai, Rubenstein, Chen, et al., 2020a; Wilson & Fudge, 1984). The frequency of these different social contexts varies both across and within *Nicrophorus* species. For example, in *N*. *vespilloides*, offspring can develop in any of the above social contexts, from biparental to uniparental to communal, or even without parents (Ma et al., 2022; Schrader et al., 2015a, 2015b; Smiseth et al., 2005). Wilson and Fudge (1984) found that mid‐larval stages in *N*. *orbicollis* and *N*. *defodiens* were more likely to have a single parent present (48% and 60%, respectively) than have biparental care (16% and 18%, respectively). Even in the laboratory, when *N*. *orbicollis* parents were allowed to “choose,” only 66% remained biparental (Benowitz & Moore, 2016). Indeed, *N*. *orbicollis* in the laboratory produce fewer offspring over their lifetime when breeding biparentally compared with beetles breeding uniparentally, with uniparental females raising the largest number of offspring over a lifetime (Smith et al., 2017). Parents can be redundant (Müller et al., 1998; Smiseth et al., 2005); for example, *N*. *orbicollis* males often reduce their activity and duration of care when females are present, but will transition to a female‐like state when the female is removed (Benowitz & Moore, 2016; Fetherston et al., 1994; Moss & Moore, 2021; Rauter & Moore, 2004; Smith et al., 2014). Consequently, offspring outcomes do not depend on the number or sex of the parent because parents trade off activities when both are present and the other parent compensates when one is gone (Bartlett & Ashworth, 1988; Parker et al., 2015; Smiseth et al., 2005; Smiseth, Musa, & Moore, 2006a).

Uniparental male *N*. *orbicollis* typically do not compensate fully for female loss or absence (Creighton et al., 2015) and as a result they successfully raise fewer offspring over a lifetime compared with uniparental females (Smith et al., 2017). Thus, rather than biparental care evolving as a backup plan, it may evolve as a division of labor where females provide most of the direct larval care, and the male's role is primarily carcass and larval defense (Scott, 1990; Trumbo, 1991, 2007), and switch to direct interactions with the larvae if the female is absent. Males may remain with the family because the opportunity to find additional carcasses is low, carcasses provide nutrition to males, and intra‐ and interspecific competition for carcasses is high (Chemnitz et al., 2017; Hopwood et al., 2015; Keppner & Steiger, 2021; Scott, 1990). That male body size influences the decision to stay at the carcass provides some support for this hypothesis. For example, *N*. *orbicollis* abandoned larvae earlier than small males (Smith et al., 2014) and smaller males breed biparentally more often than larger males (Hopwood et al., 2016a), presumably because larger males are better able to compete for new carcasses.

Guarding by parents is critical to larval survival, and appears to be the main role for the male when both parents are present (Scott, 1998; Trumbo, 1991). There are multiple threats to larvae, including competitors, parasites, and predators. Brood guarding against competitors has been hypothesized to be the primary force for a prolonged residency by one or both parents (Eggert & Müller, 1997), as parents guard even in species where larval survival is higher without parents present (e.g., *N*. *pustulatus*; Capodeanu‐Nägler et al., 2016; Rauter & Moore, 2002). Flies and nematodes strongly reduce the reproductive success of burying beetles (Sikes, 1996; Trumbo & Fiore, 1994; Wang & Rozen, 2019; Wilson, 1983), particularly in the absence of phoretic mites (Sun & Kilner, 2020; Wilson, 1983; Wilson & Knollenberg, 1987), though mites can also negatively impact larvae (De Gasperin & Kilner, 2016). Other burying beetles can usurp the brood ball and often kill larvae that are present and then start a new brood (Trumbo, 1990a, 2007). The threat imposed by congeners likely explains why species like *N*. *vespilloides* have evolved recognition mechanisms that allow discrimination between conspecific intruders and breeding partners (Steiger & Müller, 2010; Steiger, Peschke, et al., 2007a).

Guarding behavior can also deter predators, yet predation is relatively unexplored as an evolutionary mechanism that impacts parental care in *Nicrophorus*. Predation has a strong impact on parental care variation and evolution across vertebrate and invertebrate taxa (Ghalambor & Martin, 2002; Martin et al., 2000, 2011; Tallamy, 1984; Wilson, 1975). Both larvae and their parents are eaten by many foraging organisms (Coutts et al., 1973; Farriester et al., 2021; Jurzenski & Hoback, 2011; Offner et al., 2021; Potticary, Belk, et al., 2024; Reed, 1958; Scott, 1990). In the nest, parents may use anal exudate to deter predators and ants (Lindstedt et al., 2017). Adult *Nicrophorus* also decrease predation risk to themselves in several ways. Adults generally have orange and black aposematic coloration, produce anal exudate, and diurnal species like *N*. *tomentosus* may mimic bumblebee coloration and flight (Heinrich, 2012). However, the role of predators in the *Nicrophorus* system remains understudied.

### 2.5 Post‐parenting development: wandering larvae, pupae, and eclosion

Larvae disperse from the brood ball and enter a short period of wandering to find a location to bury themselves and form a chamber in which they pupate (Table 1). While post‐hatching parental care on the carcass can allow larvae to grow large quickly (see Larval stages), larger larvae of *N*. *orbicollis* and *N*. *sayi* take longer to develop once they have departed the carcass than smaller larvae (Benowitz, Amukamara, et al., 2019a; Potticary, Cunningham, et al., 2024). Following the pupal stage, individuals eclose into their adult form as a teneral adult. Teneral adults can remain underground or emerge from the soil, although they are sensitive to disturbance at this stage because their exoskeleton has not yet hardened, and they have not attained adult coloration. It is rarely reported how long it takes for adults to reach sexual maturity after eclosion.

Burying beetles can enter a seasonal diapause in prepupal, pupal, or adult stages (Anderson, 1982b; Anduaga, 2009; Pukowski, 1933; Smith, 2002), though some species like *N*. *nigrita* do not appear to enter a seasonal diapause (Sikes, 1996), and others can enter diapause in multiple developmental stages like *N*. *vespillo* (Meierhofer et al., 1999). Adults of some species also enter a reproductive diapause; for example, *N*. *quadripunctatus* and *N*. *nepalensis* have a summer reproductive diapause when temperatures are beyond a certain threshold (Hwang & Shiao, 2011; Nisimura et al., 2002). Interestingly, even within the same species, different populations of burying beetles may exhibit variation in reproductive diapause timing due to local adaptation to their specific environments. For example, there is evidence that populations of *N*. *nepalensis* breed in the winter at lower elevation, year‐round at high elevations, and in the summer at high latitudes (Tsai, Rubenstein, Fan, et al., 2020b). These differences in reproductive phenology were attributed to local adaptation in reproductive photoperiodism rather than phenotypic plasticity. Little is known about how seasonal or reproductive diapause behavior is mediated in *Nicrophorus* beyond that species vary in whether, and when, they enter diapause.

### 2.6 Adult ecology

Despite being a small and specialized group, *Nicrophorus* species have been documented across a wide variety of habitat types, from forest and grassland to desert and bogs (Figure 5; Appendix S1), and often have large ranges that encompass a diversity of social and ecological contexts. The species richness and abundance of *Nicrophorus* increase in more temperate habitats, with the greatest diversity observed at northern latitudes and higher elevations (Sikes & Venables, 2013; Trumbo, 1990c). Lower diversity or absence of burying beetles in warmer latitudes and lower elevations is thought to result from competition imposed by other necrophilous species like ants, flies, and other Coleoptera (Chan et al., 2019; Cornaby, 1974; Scott et al., 1987; Stone et al., 2021; Sun et al., 2014; Tsai, Rubenstein, Chen, et al., 2020a; von Hoermann et al., 2020). Human disturbance and climate change provide examples of temperature change that may alter range limits of burying beetles through their effects on the competitive community. For example, deforestation increased daily temperature at elevations used by *N*. *nepalensis*, which enhanced the competitiveness of blowfly maggots and led to a higher failure rate of carcass burial (Chan et al., 2023).

Habitats associations, seasonal and daily activity of adult *Nicrophorus*, are thought to reflect an evolved response to suitable breeding conditions. Consequently, *Nicrophorus* are often hypothesized to show temporal or spatial partitioning of habitats to avoid competition for limiting carrion resources (Anderson, 1982b; Burke et al., 2024; Otronen, 1988; Wettlaufer et al., 2021). Species can also be partitioned by elevation; for example, *N*. *nepalensis* occupies distinct elevational breadths in China (Liu et al., 2020). Few species have been demonstrated to prefer a single habitat type (Garfinkel & McCain, 2023; Lomolino et al., 1995), particularly when habitat preferences are documented across their range. For example, *N*. *vespilloides* have nearly circumnavigated the globe, from Europe through Asia and into western Canada (Kocárek, 2001; Ohkawara et al., 1998; Sikes et al., 2016; Sun et al., 2020), and populations differ in their habitat preferences across this range (Aleksandrowicz & Komosinski, 2005; Katakura & Ueno, 1985; Kozminykh & Esyunin, 1994; Sikes et al., 2016).

Habitats differ in their structure, abiotic conditions, and abundance and diversity of necrophilous insects and scavengers (De Jong & Chadwick, 1999; Dekeirsschieter, Verheggen, Haubruge, & Brostaux, 2011b; Katakura & Ueno, 1985; Trumbo, 1990c; Trumbo & Bloch, 2000; Tsai, Rubenstein, Fan, et al., 2020b), all of which influence the degree of competition for breeding resources. Variation in *Nicrophorus* habitat preference across a range could reflect local adaptation or flexible responses to prevailing environmental conditions. Moreover, reproductive state can influence habitat preference, as some species are captured in a broader range of habitats than are used for breeding (Smith & Heese, 1995). For example, *N*. *americanus* has greater reproductive success in forests than fields (Lomolino & Creighton, 1996) but forages in both (Creighton & Schnell, 1998).

Daily and seasonal activity patterns differ across *Nicrophorus* species (Figure 5). On ecological timescales, variation in activity periods reflects flexible responses to social or ecological conditions. Over evolutionary time, activity periods are expected to reflect evolved differences in thermal tolerance and/or environments created by species interactions (Benowitz, Amukamara, et al., 2019a; Cook et al., 2019; Merrick & Smith, 2004; Quinby, Belk, & Creighton, 2020a; Scott, 1998; Trumbo, 1990c; Wilson et al., 1984). For example, *N*. *nepalensis* differ in their periods of reproductive activity depending on what mountain range and elevation they occupy, even though thermal tolerance is similar across populations, reflecting local adaptation to abiotic conditions (Tsai, Rubenstein, Fan, et al., 2020b). Across a latitudinal gradient, populations of *N*. *orbicollis* show local adaptation to temperature in their willingness to initiate breeding and brood sizes (Quinby, Belk, & Creighton, 2020a). Ecological pressure arising from the social environment, particularly from competitors, is also expected to influence *Nicrophorus* activity (Anderson, 1982b) and social behavior (Sun et al., 2014).

Food is not thought to be limiting for adult *Nicrophorus*, as species are less discerning in their feeding resources than their breeding ones (Figure 2). For this reason, competition for breeding resources is thought to influence seasonal activity. Central to the hypothesis that competition influences the evolution of activity patterns is the assumption that competition is predictable to some extent. How can predictable activity periods arise from a seemingly unpredictable and ephemeral resource? One possibility is that activity periods are driven by the strength of competition imposed by the necrophilous community, perhaps with temperature as a cue. Temperature and competition are tightly aligned; the phenology of necrophilous species differs based on season and habitat (Anderson & Peck, 1985; Wilson et al., 1984). Alternatively, *Nicrophorus* may time their activity to capitalize on periods where carrion can be anticipated (e.g., salmon spawning; Hocking et al., 2006, 2007). Among‐population variation in factors like temperature and the composition of the necrophilous community has the potential to drive local adaptation in *Nicrophorous* beetles. For example, two recently separated populations of *N*. *vespilloides* show evidence for local adaptation in response to variation in local burying beetle guilds (Sun et al., 2020). It remains unknown whether other kinds of ecological pressures, like parasitism and predation, influence *Nicrophorus* activity and whether populations are locally adapted to these pressures.

## 3 EVOLUTION OF PARENTAL CARE IN *NICROPHORUS*

Burying beetles have attracted the attention of ecologists, geneticists, behavioral and evolutionary biologists because of their extraordinary parenting behavior. As such, *Nicrophorus* has provided insights into how parental care may evolve, the changes required, and the selection pressures driving such changes.

### 3.1 Behavioral precursors for parental care

Selection for a trait can only occur after that trait, or its components, exists. Because behavior is context‐dependent, ecology determines both the opportunities for behaviors to be expressed and the availability of those behaviors to selection. For this reason, complex behaviors are expected to evolve when behavioral precursors are expressed in a new context (Moore & Benowitz, 2019; Tallamy, 1984; West‐Eberhard, 2003). To investigate this hypothesis, we looked for the existence of behavioral precursors and scenarios where these behavioral precursors are expressed in the situation into which they will eventually be co‐opted. In the case of parental care, this requires that behavioral precursors transition from a historically non‐parental function to a parental care function (Moore & Benowitz, 2019). Below, we describe potential behavioral precursors for the parenting behaviors in *Nicrophorus* described above, mechanistic evidence for co‐option, and conclude with a hypothesized order of co‐option for parental care of *Nicrophorus*.

#### 3.1.1 Nesting resource acquisition: carcass seeking and preference

Carrion beetles broadly use sulfur‐containing volatiles to locate carrion (Cammack et al., 2015; Trumbo & Newton, 2022), and non‐parental silphines also use carcasses at specific stages of decay (Anderson & Peck, 1985; Byrd & Castner, 2001; Lis et al., 2024; Martin et al., 2020; Matuszewski et al., 2010; Müller et al., 2024; Ratcliffe, 1996; Trumbo & Newton, 2022; Watson & Carlton, 2003, 2005). Attraction to small fresh carcasses is not unique to *Nicrophorus* in the Silphinae; *Necrophila* are also attracted to fresh carcasses (Ito, 2020, 2022; Ito et al., 2023; Trumbo & Dicapua, 2021). While the carcass decomposition stage used for breeding sometimes differs across Silphinae, the main takeaway is that the ability to distinguish and find carcasses at different stages of decomposition is common to all carrion beetles. Thus, carcass finding behavior in burying beetles likely involved an evolutionary change in the preference for the stage of carcass decomposition rather than the de novo ability to interpret stage‐specific cues of carrion decomposition during searching. This change in preference may have been due to selection imposed by necrophilous predators or competitors, which increase after the early stages of decomposition (Byrd & Castner, 2001; Kočárek, 2003; Matuszewski et al., 2008).

#### 3.1.2 Securing the carcass: burial and carcass preparation

Burial behavior requires not only the act of burying but also a preference for concealing the resource, with the preference for carcasses underground preceding burial behavior itself. One possibility is that the preference for carcasses underground arose from either finding or dragging carcasses to pre‐existing holes, from which burial behavior secondarily evolved. Representatives from nearly every group of *Nicrophorus*, including *N*. *concolor*, *N*. *defodiens*, *N*. *guttula*, *N*. *hybridus*, *N*. *investigator*, *N*. *orbicollis*, and *N*. *marginatus*, have been observed to either drag carcasses into mammal burrows or to breed using carcasses discovered in burrows (Ito, 2021; Parmenter & MacMahon, 2009; Scott, 1990; Smith, Bonilla, et al., 2000a; Wilson & Fudge, 1984). *Nicrophorus pustulatus* does not bury breeding resources that are typically discovered underground, that is, reptile eggs, but will bury mammals (Smith et al., 2007). While *P*. *morio* does not bury carcasses experimentally placed on the ground (Peck, 1982; Suzuki & Nagano, 2006b), *P*. *morio* will use buried carcasses (Trumbo et al., 2001), and *P*. *plagiatus* finds and breeds on carrion buried at 30 cm (Zou et al., 2022). If breeding on buried carrion provides a substantial fitness benefit, then a preference for buried carrion could have been extended to the active burying of carrion in *Nicrophorus*.

Components of carcass preparation like removing the external covering, guarding, and creation of incisions are exhibited by relatives of *Nicrophorus* and may have been co‐opted from aspects of adult foraging behavior. For example, removing fur is thought to be a tactic for hunting fly larvae (Ratcliffe, 1972). Multiple non‐parental silphines also demonstrate these behaviors. *Ptomascopus morio* removes fur, guards, opens feeding holes where larvae aggregate, and makes abdomen movements on the carcass like they are depositing exudate, yet they do not deposit exudate or ball up the carcass, and feeding holes do not improve larval growth (Suzuki & Nagano, 2006b; Trumbo et al., 2001). *Necrodes surinamensis* strips fur from carrion, and larvae form communal aggregations in incisions in multiple *Necrodes* species (Lis et al., 2024; Ratcliffe, 1972). Therefore, these behaviors likely existed in the most recent common ancestor of *Necrodes* and *Nicrophorus* and were co‐opted into parenting by *Nicrophorus*.

Parental mediation of the microbial community on carcasses involves both the mechanisms of mediation—the compounds and microbes—and the behavior of exuding these compounds in the appropriate context. It has been broadly hypothesized that the compounds *Nicrophorus* parents apply to carcasses were co‐opted from common mechanisms of personal immunity and digestive function used by insects that frequent microbe‐rich environments like carrion (Otti et al., 2014; Van Herreweghe & Michiels, 2012). Lysozymes and small antimicrobial peptides, like those secreted by *Nicrophorus* parents, are broadly reported across taxonomic groups (Bulet et al., 1999; Hall et al., 2011; Van Herreweghe & Michiels, 2012; Zasloff, 2002). The lysozymes that parents apply to carcasses are also upregulated in *Nicrophorus* following infection and immunosuppression occurs during the provisioning of parental care, consistent with a trade‐off between personal immunity and carcass preparation (Cotter et al., 2013; Cotter & Kilner, 2010; Palmer et al., 2016; Reavey et al., 2014). Components of the secretions produced by parent and larva *Nicrophorus*, like *Yarrowia* fungi (Vogel et al., 2017), have been detected in other non‐parental silphines (reviewed in Körner et al., 2023). *Necrodes surinamensis* produce anal exudate for defense that has antimicrobial properties (Eisner & Meinwald, 1982; Hoback et al., 2004) and *Necrodes littoralis* adults and larvae use anal exudates to create a feeding matrix that benefits larval development (Lis et al., 2024; Matuszewski & Mądra‐Bielewicz, 2021). *Necrophila japonica* also places exudate on carcasses (Ito et al., 2023). The use of anal exudate in defense against predators and intruders is common in silphines (Lindstedt et al., 2017; Ratcliffe, 1972). Trumbo and Sikes (2021) hypothesized that the use of anal exudate as a defense against predation (containing microbiota from the digestive tract and common components of the insect immune system (Cotter et al., 2013; Kaltenpoth & Steiger, 2014; Miller et al., 2019; Shukla, Vogel, et al., 2018b; Steiger et al., 2011; Wang & Rozen, 2017) enabled the evolution of carcass preparation. Predation of both larvae and parents occurs on carcasses. As such, excreting exudate as an anti‐predator device on carcasses may have been an initial step in the evolution of carcass preparation, and then the use of exudate to hide the carcass by reducing the emission of volatiles evolved secondarily.

#### 3.1.3 Larval stages on the brood ball: direct care and associations with larvae

The evolution of interactions between parents and offspring is complex. A transition to expressing parental care towards larvae requires adults to recognize and tolerate a larva‐like form. Once tolerance has evolved, then recognition of larval begging, and regurgitation of carrion and other social fluids (Hakala et al., 2023) by connecting mouthparts is possible. Because larval care in *Nicrophorus* can involve multiple adults, post‐hatching care can also require tolerance between adults at the breeding carcass. The ability to recognize larvae and tolerate other adults in the presence of larvae may have been co‐opted from components of parental foraging behavior. Foraging *Nicrophorus* and relatives are well‐known to hunt larvae on a diversity of substrates (see Section 2.1.1). Post‐hatching parental care primarily involves an inhibition of infanticide at a particular time rather than the ability to recognize a particular set of larvae, which may indicate that attraction to larvae, albeit as food, was a behavioral precursor for direct parental care. Indirect support for this idea comes from the observation that many *Nicrophorus* parents will accept any larvae that appear at the correct time and congregate in the larval cavity, and larvae will accept any parent, regardless of species (Benowitz et al., 2015; Bladon et al., 2023; Eggert & Müller, 2000; Müller & Eggert, 1990; Oldekop et al., 2007; Scott, 1997; Smith & Belk, 2018b; Trumbo, 1994), although there are some exceptions to this (Capodeanu‐Nägler, Ruiz de la Torre, et al., 2018b; Smith & Belk, 2018b). Furthermore, parents are not adept at removing blowfly larvae that appear in later stages of the parental care cycle, that is, once their larvae have appeared (Springett, 1968; Wilson, 1983).

That genes associated with adult feeding behavior have also been associated with the acceptance of larvae during temporal kin recognition (Cunningham et al., 2016; Potticary, McKinney, et al., 2023a) provides support for the idea that aspects of foraging behavior have been co‐opted to enable the evolution of larval recognition and affiliation. Moreover, the evolution of multiple individuals exhibiting parental care requires a transition from competition to cooperation between adults. A transition to parenting that involves more than one individual requires parents tolerating one another and excluding other beetles in response to a particular resource type—or ability to differentiate between partner and non‐partner (e.g., differentiating based on cuticular hydrocarbons; Steiger, Peschke, et al., 2007a). Foraging *Nicrophorus* are not aggressive to other *Nicrophorus* on unsuitable breeding substrates, and high blowfly maggot activity on breeding carcasses can induce *N*. *nepalensis* to transition from intraspecific competition to cooperation against blowflies (Chen et al., 2020). Together, these data support the hypothesis that foraging behaviors on carcasses have been co‐opted and modified to produce contemporary parental care.

### 3.2 Mechanisms of parental care

Novel behaviors are thought to evolve when behavioral precursors and their underlying mechanisms are expressed in new situations or different ways (Cunningham et al., 2017; Moore & Benowitz, 2019; Tallamy, 1984; West‐Eberhard, 2003). Moreover, the creation of a complex behavior like parental care also requires mechanisms that can link component behaviors together. Such integration allows for suites of behaviors to be expressed together when an organism interacts with a specific context. For *Nicrophorus*, what is the mechanistic evidence that behavioral precursors have been co‐opted and linked to generate complex parental care?

*Nicrophorus* species have a small genome (~200 MB; Cunningham et al., 2015) and few chromosomes (1*N* = 6 + X; Smith, 1953). This has facilitated the development of molecular tools including transcriptomes (Ayala‐Ortiz et al., 2021; Palmer et al., 2016; Parker et al., 2015; Vogel et al., 2017; Won et al., 2018) and genomes (Benowitz et al., 2017; Cunningham et al., 2015) with more genomes to come (Benowitz, pers. comm., Shen, pers. comm.). Genetic data provide support for the hypothesis that behavioral precursors expressed in non‐parental contexts were co‐opted into parenting. One of the best examples is the relationship between feeding and parental care. It has been hypothesized that the systems influencing self‐feeding and parental care are coregulated and that parental care evolves through co‐option and modification of feeding systems (O'Rourke & Renn, 2015; West‐Eberhard, 2003). Gene expression of neuropeptide F receptor, a pathway associated with the motivation to eat, decreases when parents are feeding larvae in *N*. *vespilloides* (Cunningham et al., 2016). In *N*. *vespilloides*, several genes involved in feeding are differentially expressed in both sexes in the transition to parenting from non‐parenting (Parker et al., 2015) and are associated with variation of active larval care (Benowitz, McKinney, et al., 2019b). The gene *takeout* is differentially expressed during a transition to parenting in *N*. *vespilloides* (Parker et al., 2015) and *N*. *orbicollis* (Moss et al., 2022), and has been associated with coordinated feeding and time of day across a variety of insect taxa, supporting the idea that *takeout* has been co‐opted for parenting (Moore et al., 2010; Potticary, McKinney, et al., 2023a). Neuropeptides associated with parenting states in female *N*. *vespilloides* are also associated with feeding, social interactions, aggression, and resource defense in non‐parental contexts (Cunningham et al., 2017). Moreover, similar mechanisms can influence the expression of the behavioral components of parental care in both sexes of *N*. *orbicollis* and *N*. *vespilloides* (Benowitz et al., 2017; Cunningham et al., 2017; Moss et al., 2022; Parker et al., 2015). That these mechanisms are not sex‐specific provides indirect support for the hypothesis that parental behaviors evolved from behaviors common to both sexes, such as foraging behavior.

*Nicrophorus* parents show a suite of behaviors that must be expressed together relative to a particular functional context—the presence of a suitable breeding resource. Carcass availability is unpredictable, and mating can occur on or off the carcass. Thus, physiological transitions to a parental care state require a flexible mechanism that is responsive to an unpredictable resource and can impact multiple behaviors simultaneously. Regulation of these behavioral components could be accomplished hormonally by JH (Panaitof et al., 2004; Scott et al., 2001; Trumbo et al., 1995). JH biosynthesis is sensitive to nutritional cues across insect taxa, and thus, the involvement of JH in a transition to parental care relative to a feeding resource is intuitive. JH has been broadly implicated in both care and feeding behaviors, and mediating transitions between them, across insects (O'Rourke & Renn, 2015). In *N*. *orbicollis*, JH surges correspond to both non‐parenting feeding roles and feeding that occurs in a parental context. JH increases when the beetles emerge as adults from the soil and begin the search for food during sexual maturation, as well as in parental contexts, such as upon discovery and assessment of a breeding carcass, and when the young larvae arrive at the carcass (Panaitof et al., 2004; Trumbo, 1997; Trumbo et al., 1995). It is unclear whether JH ancestrally had gonadotropic and non‐gonadotropic functions, or if a gonadotropic function in *Nicrophorus* was co‐opted to integrate parenting behaviors across the reproductive cycle (Trumbo, 2018).

Indirect and direct care behaviors have been associated with JH in *Nicrophorus* of both sexes. Juvenile hormone influences the transition from mating to a parental care state in *N*. *vespilloides* (Engel et al., 2016), regulation of direct provisioning of larvae in *N*. *vespilloides*, *N*. *orbicollis*, and *N*. *pustulatus* (Engel et al., 2016; Trumbo & Rauter, 2014), and potentially the upregulation of lytic activity in anal exudates that are involved in carcass preparation in *N*. *vespilloides* (Cotter & Kilner, 2010). Males of *N*. *orbicollis* and *N*. *vespilloides* are known to show a subset of parental care behaviors when the female parent is present but will transition to a full suite of parental care behaviors when the female is removed, produced through coordinated changes in many genes such that transcription profiles become more similar to parenting females (Benowitz & Moore, 2016; Moss & Moore, 2021; Parker et al., 2015). Concomitant with an involvement in organizing parental care behaviors, uniparental *N*. *orbicollis* males show higher JH titers than males breeding biparentally (Panaitof et al., 2004; Trumbo & Rauter, 2014). Overall, the mechanisms supporting parenting in *Nicrophorus* are complex but provide evidence for the co‐option of genes that underlie behavioral expression in other contexts, like feeding, and hormonal integration of these behaviors relative to an ephemeral resource.

### 3.3 Hypothesized evolutionary trajectory for parental care in *Nicrophorus*

Complex behaviors like parental care are constructed of multiple behaviors that can function independently or together as an integrated suite. Stabilizing selection is expected to produce greater integration between behaviors, leading to initially disparate components becoming phenotypically, developmentally, and genetically linked (Cheverud, 1996). Integration requires mechanisms that can link behaviors together, and as a result, integration is expected to limit flexibility and evolvability across timescales (Coss & Goldthwaite, 1995; Kauffman & Levin, 1987; Wagner & Altenberg, 1996). Integration can occur within behavioral modules that accomplish specific tasks—for example, carcass preparation (Duarte et al., 2021)—or at the level of a complex behavior as a whole (i.e., parental care). Thus, understanding how component behaviors relate to each other and how quickly they can be lost may provide insight into how complex behaviors like parenting are assembled.

Hypotheses about the early stages of parental care evolution in *Nicrophorus* have been informed by comparisons between *Nicrophorus* spp. and *P*. *morio*. *Ptomascopus* is not the closest extant genus to *Nicrophorus*, a category restricted to the virtually unstudied and monotypic genus *Eonecrophorus*, but *Ptomascopus* is the sister genus to the clade containing *Eonecrophorus* and *Nicrophorus*. *Ptomascopus morio* parental care behaviors represent a subset of the parental behaviors of *Nicrophorus* and include (a) preference for breeding on small fresh carcasses, (b) defense of the carcass and brood, (c) partial carcass preparation, including some shaving and cutting of incisions, (d) abdomen movements that look like exudate laying, without secretion deposition, (e) tolerance of larvae, and (f) one or multiple other *Ptomascopus* adults on or near the carcass. *Ptomascopus plagiatus* will also breed on carrion found underground (Zou et al., 2022), as will *P*. *morio* when it acts as a brood parasite of *Nicrophorus* (Trumbo et al., 2001). Little is known about the behavior of other *Ptomascopus* species besides *P*. *morio*. There are two main possibilities for how the parental care of *Ptomascopus* and *Nicrophorus* relate. *Ptomascopus* may be an example of how early parenting looked in ancestral Nicrophorini. However, behaviors of extant species are not necessarily representations of early evolutionary stages because behaviors are routinely deleted or subsumed by new functions over time (West‐Eberhard, 2003). Thus, an alternative explanation is that *Ptomascopus* has lost components of parental care that were historically present in the most recent common ancestor of *Nicrophorus* and *Ptomascopus*. Regardless of whether *Ptomascopus* demonstrates early care or has lost components of care, applying an integration perspective can provide insight into the history of co‐option in this group. An integration perspective predicts that behaviors that are shared by *Nicrophorus* and *Ptomascopus* are more developmentally and phylogenetically entrenched, reflecting either stronger selection and/or inheritance from their common ancestor.

It is probable that some of the earliest innovations in nicrophorine parenting were guarding, carcass preparation, recognition, and tolerance of larvae. Guarding is apparent across *Nicrophorus* and in *P*. *morio*, including species that breed on small, defensible resources, and also species like *N*. *investigator* that sometimes breed on carcasses that are too large to bury (Hocking et al., 2006). *Nicrophorus pustulatus* parents guard larvae even though larvae are nutritionally independent (Capodeanu‐Nägler et al., 2016). *Ptomascopus morio* not only guards the carcass from competitors but also defends against predators (Suzuki & Nagano, 2006b). Other staphylinids also demonstrate guarding behavior, even in post‐hatching stages (Wyatt & Foster, 1989), perhaps indicating that guarding behavior occurred early in the evolution of parenting. That adults recognize and approach larvae‐like forms is likely to be ancestral to the Nicrophorini, based on the widespread prevalence of silphines adults predating larvae (see Section 2.1.1). If this is the case, tolerance of, rather than preying upon, larvae on the carcass must have been an early step in the transition to extended parental care.

Carcass preparation has been observed in all *Nicrophorus* species studied to date. Activities like shaving the carcass and cutting incisions are components of foraging ecology that are present in many Silphinae and other staphylinids (see Section 2.1.1). Exudate is produced as an anti‐predator mechanism across taxa, and early carcass preparation could have emerged as a byproduct of parents guarding carcasses from potential intruders. Moreover, *P*. *morio* demonstrates abdomen movements on the carcass that resemble the exudate‐laying behavior of *Nicrophorus*, although they do not place secretions on the carcass. This behavior resembles other systems where “vestigial” behaviors outlast the morphological traits they accompanied (Coss et al., 1999; Coss & Goldthwaite, 1995; Rayner et al., 2022), which may indicate that *P*. *morio* historically prepared carcasses and has lost components of carcass preparation over time.

Burial is the most variable of the indirect care behaviors across *Nicrophorus* (Figure 5). *Ptomascopus morio* and other non‐parental silphines do not bury at all. Despite this, all *Nicrophorus* and *P*. *plagiatus* will use carcasses that they find that are already underground, and *P*. *morio* will parasitize *Nicrophorus* broods that are buried (Suzuki & Nagano, 2006a; Trumbo et al., 2001). Based on this variability, burial may be a more recent innovation in the evolution of parental care.

Direct provisioning of larvae is likely to be the most recently evolved parental care behavior. There is extreme variation in the flexibility and duration of larval care, as well as the dependence of larvae on parental regurgitations across taxa (Figure 5). Direct provisioning could have arisen from the parents partially digesting the carcass and providing a “soup” from which the larvae feed in the cavity. This puts parents and larvae in direct contact from which both mouth‐to‐mouth contact and begging can evolve. In other insect species that beg, such as honeybees and ants, trophallaxis appears to have arisen prior to begging. During experimental evolution, the propensity of larvae to beg is one of the first behaviors that is lost when direct care is removed for multiple generations in *N*. *vespilloides* (Bladon et al., 2023), perhaps suggesting that larval begging is one of the more recently evolved behaviors in this taxa.

## 4 CONCLUSION AND FUTURE DIRECTIONS

Burying beetles provide a rich experimental system for understanding the evolution of social behaviors like parental care. The strength of the research community investigating burying beetle biology and ecology has provided a wealth of information on which to build. There is clearly considerable variation that can be leveraged to understand how various levels, from physiology to development to genetics to ecology to phylogeny, influence the evolution of complex traits such as parenting. In addition, there are many areas we have tried to highlight that would benefit from more research, particularly documenting inter‐ and intraspecific variation in behavior and ecology via the inclusion of more *Nicrophorus* species that have received little attention to date. Study of the virtually unknown sister taxon to *Nicrophorus*, *Eonecrophorus*, and the subgenus *Necroxenus*, should be high priorities for understanding the origin of parental care in the genus.

## AUTHOR CONTRIBUTIONS

**Ahva L. Potticary:** Conceptualization (lead); data curation (lead); investigation (lead); project administration (lead); visualization (lead); writing – original draft (lead); writing – review and editing (lead). **Mark C. Belk:** Data curation (supporting); investigation (supporting); writing – review and editing (supporting). **J. Curtis Creighton:** Data curation (supporting); investigation (supporting); writing – review and editing (supporting). **Minobu Ito:** Data curation (supporting); investigation (supporting); writing – review and editing (supporting). **Rebecca Kilner:** Data curation (supporting); investigation (supporting); writing – review and editing (supporting). **Jan Komdeur:** Data curation (supporting); investigation (supporting); writing – review and editing (supporting). **Nick J. Royle:** Data curation (supporting); investigation (supporting); visualization (supporting); writing – review and editing (supporting). **Dustin R. Rubenstein:** Data curation (supporting); investigation (supporting); writing – review and editing (supporting). **Matthew Schrader:** Data curation (supporting); investigation (supporting); writing – review and editing (supporting). **Sheng‐Feng Shen:** Data curation (supporting); investigation (supporting); writing – review and editing (supporting). **Derek S. Sikes:** Data curation (supporting); investigation (supporting); visualization (supporting); writing – review and editing (supporting). **Per T. Smiseth:** Data curation (supporting); investigation (supporting); writing – review and editing (supporting). **Rosemary Smith:** Data curation (supporting); investigation (supporting); writing – review and editing (supporting). **Sandra Steiger:** Data curation (supporting); investigation (supporting); writing – review and editing (supporting). **Stephen T. Trumbo:** Data curation (supporting); investigation (supporting); writing – review and editing (supporting). **Allen J. Moore:** Conceptualization (supporting); data curation (supporting); funding acquisition (lead); methodology (supporting); project administration (supporting); resources (lead); visualization (supporting); writing – original draft (supporting); writing – review and editing (supporting).

## FUNDING INFORMATION

ALP was supported by a United States Department of Agriculture (USDA) cooperative agreement to AJM.

## CONFLICT OF INTEREST STATEMENT

The authors declare no conflict of interest.

## Supporting information


Appendix S1.

## Data Availability

Data used to support figures are provided in Dryad. A link to these data for the review process: https://datadryad.org/stash/share/0Jl1VN5cpvRWl73vc0tGcYDMbOckQRJ2w9uFFhA6LTQ.
