# Correction to “Revisiting the Ecology and Evolution of Burying Beetle Behavior (Staphylinidae: Silphinae)”

**DOI:** 10.1002/ece3.72393

**Published:** 2025-10-27

Potticary, A. L., M. C. Belk, J. C. Creighton, M. Ito, R. Kilner, J. Komdeur, N. J. Royle, D. R. Rubenstein, M. Schrader, S.‐F. Shen, D. S. Sikes, P. T. Smiseth, R. Smith, S. Steiger, S. T. Trumbo, and A. J. Moore. 2024. “Revisiting the Ecology and Evolution of Burying Beetle Behavior (Staphylinidae: Silphinae).” *Ecology and Evolution* 14: e70175. https://doi.org/10.1002/ece3.70175.

In the published article, a temporary URL was included in the Data Availability Statement. The data can now be accessed at the permanent URL: https://datadryad.org/dataset/doi:10.5061/dryad.nzs7h4501.

We apologize for this error.

