# Supplementary material for: Revisiting the ecology and evolution of burying beetle behavior (Staphylinidae: Silphinae)
Source: Ecol Evol. 2024 Aug 20;14(8):e70175. doi: 10.1002/ece3.70175 (PMC11336061; doi:10.1002/ece3.70175)
Supplement: Supplementary file 1 — Appendix S1. [file ECE3-14-e70175-s001.docx]

| **Species:**  **N = *Nicrophorus***  **P = *Ptomascopus*** | **Embryonic period** | **Hatch to dispersal** | **Dispersal to eclosion** | **Eclosion to sexual maturity** | **Offspring dependency on post-hatching care** | **Burial depth** | **Avg. body size** | **Habitat** | **Daily activity** | **Seasonal activity** |
| --- | --- | --- | --- | --- | --- | --- | --- | --- | --- | --- |
| *N. americanus* |  |  |  |  | [1] | [2] | [1] | [2-8] | [2, 6, 7, 9-11] | [2] |
| *N. antennatus* |  |  |  |  |  |  | [1] | [12, 13] | MB | [14] |
| *N. apo* |  |  |  |  |  |  | [1] | [15] |  |  |
| *N. argutor* |  |  |  |  |  |  | [1] | [16] |  |  |
| *N. basalis* |  |  |  |  |  |  |  |  |  |  |
| *N. carolina* |  |  |  |  | ST | [17] | [1] | [18-21] |  | [22] |
| *N. charon* |  |  |  |  |  |  | [1] |  |  |  |
| *N. chilensis* |  |  |  |  |  |  |  | [23] |  | [23] |
| *N. concolor* | [24] | [24] | [24] | [24] | MI | [25] | [1] | [26-28] | [24, 29] | [27] |
| *N. dauricus* |  |  |  |  |  |  | [1] | [13] |  |  |
| *N. defodiens* | CC, RS | [30], RS | [30], RS | [31] | [32] | [18, 33-37] | [38] | [18, 21, 37, 39-41] | [18] | iNaturalist, RS, [42] |
| *N. didymus* |  |  |  |  |  |  | [1] | [43] |  |  |
| *N. distinctus* |  |  |  |  |  |  | [1] |  | DS |  |
| *N. efferens* |  |  |  |  |  |  | [44] |  |  |  |
| *N. encaustus* |  |  |  |  |  |  | [1] |  |  |  |
| *N. germanicus* |  |  |  |  |  | [45] | [1] | [12, 13] | [46] | [45] |
| *N. guttula* | MCB, RS | MCB, RS | MCB, RS |  |  | MCB | [1] | [18, 21, 43] | [18, 47] | iNaturalist, RS |
| *N. hebes* |  |  |  |  |  | [18, 48] | [49] | [18, 40, 41, 50] |  | [42] |
| *N. herscheli* |  |  |  |  |  |  |  |  |  |  |
| *N. heurni* |  |  |  |  |  |  | [1] | [15] |  |  |
| *N. hispaniola* |  |  |  |  |  |  | [51] | [51, 52] | [51] |  |
| *N. humator* |  |  |  |  |  | [45] | [1] | [12, 13, 53-57] | [53, 58] | [53, 55, 59, 60] |
| *N. hybridus* |  |  |  |  |  |  | [1] | [18, 21] | [18] | iNaturalist, RS |
| *N. insignis* |  |  |  |  |  |  |  |  |  |  |
| *N. insularis* |  |  |  |  |  |  | [1] | [15] | DS |  |
| *N. interruptus* |  |  |  |  |  | [45] | [1] | [21, 53-56, 58] | [53, 58] | [53, 55, 58] |
| *N. investigator* | RS | RS | RS |  | SS | [61-66] | [1] | [12, 13, 21, 26, 37, 39, 53-56, 63, 65, 67, 68] | [53, 58, 68] | [53, 55, 60], RS |
| *N. japonicus* |  |  |  |  |  |  | [1] | [28, 69] | DS |  |
| *N. kieticus* |  |  |  |  |  |  |  | [1] |  |  |
| *N. lethaeus* |  |  |  |  |  |  |  |  |  |  |
| *N. lunatus* |  |  |  |  |  |  |  | [1] |  |  |
| *N. maculifrons* |  |  |  |  |  | [70] | [27] | [26, 27, 67, 68, 70] | [70] | [27, 67, 70] |
|  |  |  |  |  |  |  |  |  |  |  |
| **Species:**  **N = *Nicrophorus***  **P = *Ptomascopus*** | **Embryonic period** | **Hatch to dispersal** | **Dispersal to eclosion** | **Eclosion to sexual maturity** | **Offspring dependency on post-hatching care** | **Burial depth** | **Avg. body size** | **Habitat** | **Daily activity** | **Seasonal activity** |
| *N. marginatus* | MCB | MCB | MCB |  | SS | [2] MCB | [71] | [5, 19-21, 40, 43, 72-77] | [11, 47, 76] | [20, 42, 74] |
| *N. melissae* |  |  |  |  |  |  |  | [78] |  |  |
| *N. mexicanus* | [79, 80] | [79, 81, 82] | [79-81] | [79] | [83] | [80] | [1] | [21, 43, 77, 84] |  | [43, 84] |
| *N. mongolicus* |  |  |  |  |  |  |  |  |  |  |
| *N. montivagus* |  |  |  |  |  |  | [1] |  |  |  |
| *N. morio* |  |  |  |  |  |  | [1] | [85] | [85] |  |
| *N. nepalensis* | [86], SFS | SFS | SFS | [87] | [1] | [88] | [1] | [88, 89] | [88, 89] | [87] |
| *N. nigricornis* |  |  |  |  |  |  | [1] |  |  |  |
| *N. nigrita* |  | [90] | [90] |  |  | [90] | [1] | [43, 90] | [90] | [90] |
| *N. oberthuri* |  |  |  |  |  |  | [1] |  |  |  |
| *N. obscurus* |  |  |  |  |  |  | [1] | [18, 21] | [18] | [18] |
| *N. olidus* |  |  |  |  |  |  | [1] | [43, 77, 84] |  | [43, 84] |
| *N. orbicollis* | [91, 92] | [91] | [91] |  | [32, 92, 93] | [35, 48] | [1] | [5, 18, 20, 39, 40, 50, 72, 75, 94, 95] | [18, 96] | [18, 20, 42, 94, 95, 97] |
| *N. podagricus* |  |  |  |  |  |  | [1] | [15, 78] | D.S. |  |
| *N. przewalskii* |  |  |  |  |  |  | [1] | [78] |  |  |
| *N. pustulatus* | [98-100] | [98, 100] | [100] |  | [32, 93, 100] | [36, 99] | [1] | [19, 20, 39, 40, 50, 75, 98, 101] | [11, 18, 102] | DS, KB, [42] |
| *N. quadraticollis* |  |  |  |  |  |  |  |  |  |  |
| *N. quadrimaculatus* |  |  |  |  |  |  | [1] | [43, 103] | D.S. | [43] |
| *N. quadripunctatus* | [104-106] | [105] | [105] |  |  | [70] | [1] | [26-28, 67, 68, 70, 107] | [68, 70, 108] | [27, 67, 70] |
| *N. reichardti* |  |  |  |  |  |  |  |  |  |  |
| *N. reticulatus* |  |  |  |  |  |  |  |  |  |  |
| *N. satanas* |  |  |  |  |  |  |  | [13] |  |  |
| *N. sausai* |  |  |  |  |  |  |  |  |  |  |
| *N. sayi* | [109] | [109] | [109] |  | [32] | [35, 36] | [1] | [18, 39-41, 50] | [18] | DS, KB, [42, 97] |
| *N. schawalleri* |  |  |  |  |  |  |  |  |  |  |
| *N. scrutator* |  |  |  |  |  |  | [1] | [43] |  | [43] |
| *N. semenowi* |  |  |  |  |  |  | [1] |  |  |  |
| *N. sepulchralis* |  |  |  |  |  |  |  |  |  |  |
| *N. sepultor* |  |  |  |  |  |  | [1] | [12, 13, 54, 56] |  |  |
| *N. smefarka* |  |  |  |  |  |  | [1] |  |  |  |
| *N. tenuipes* |  |  |  |  |  |  | [1] | [68] | [68] |  |
|  |  |  |  |  |  |  |  |  |  |  |
| **Species:**  **N = *Nicrophorus***  **P = *Ptomascopus*** | **Embryonic period** | **Hatch to dispersal** | **Dispersal to eclosion** | **Eclosion to sexual maturity** | **Offspring dependency on post-hatching care** | **Burial depth** | **Avg. body size** | **Habitat** | **Daily activity** | **Seasonal activity** |
| *N. tomentosus* |  |  |  |  | [32] | [18, 35, 36, 110] | [38] | [5, 18, 20, 21, 39, 40, 50, 72, 75, 94, 95, 101] | [11, 18, 96] | [42, 94, 95] |
| *N. trumboi* |  |  |  |  |  |  |  |  |  |  |
| *N. ussuriensis* |  |  |  |  |  |  |  |  |  |  |
| *N. validus* |  |  |  |  |  |  |  |  |  |  |
| *N. vespillo* |  |  |  |  | [1] | DS | [1] | [12, 13, 53-55] | [53] | [53, 55] |
| *N. vespilloides* | [111-113] | [113, 114] | [113] | [113] | [115] | [70] | [1] | [12, 13, 26, 53-55, 66-68, 70, 107, 116] | [53, 70] | [53, 55, 60, 67, 70] |
| *N. vestigator* |  |  |  |  |  |  |  | [13, 54] |  |  |
| *P. morio* | [117] | [117] | [117] |  | [117, 118] |  | [1] | [26, 27, 67] | [118] |  |

***Initials for personal communications***

DS: Derek Sikes

KB: Kyle Benowitz

MB: Max Barclay, pers. obs. to D.S.

MCB: Mark Belk

MI: Minobu Ito

RS: Rosemary Smith

SFS: Sheng-Feng Shen

iNaturalist: iNaturalist data, available in Dryad file, Potticary et al. 2024 [92]

***References***

1. Jarrett, B.J.M., et al., *Cooperative interactions within the family enhance the capacity for evolutionary change in body size.* Nature Ecology and Evolution, 2017. **1**(7): p. 0178.

2. Kozol, A.J., M.P. Scott, and J.F. Traniello, *The American burying beetle, Nicrophorus americanus: studies on the natural history of a declining species.* Psyche, 1988. **95**(3-4): p. 167-176.

3. Creighton, J.C. and G.D. Schnell, *Short-term movement patterns of the endangered American burying beetle Nicrophorus americanus.* Biological Conservation, 1998. **86**(3): p. 281-287.

4. Creighton, J.C., et al., *Effect of forest removal on the abundance of the endangered American burying beetle, Nicrophorus americanus (Coleoptera: Silphidae).* Journal of Insect Conservation, 2009. **13**: p. 37-43.

5. Lomolino, M.V. and J.C. Creighton, *Habitat selection, breeding success and conservation of the endangered American burying beetle Nicrophorus americanus.* Biological Conservation, 1996. **77**(2-3): p. 235-241.

6. Walker Jr, T.L. and W.W. Hoback, *Effects of invasive eastern redcedar on capture rates of Nicrophorus americanus and other Silphidae.* Environmental Entomology, 2007. **36**(2): p. 297-307.

7. Howard, D.R. and C.L. Hall, *Examining the management of rare insects through the lens of biotic interactions: a comparative case study of Nicrophorus americanus (Coleoptera: Silphidae) and Gryllotalpa major (Orthoptera: Gryllotalpidae).* Annals of the Entomological Society of America, 2019. **112**(3): p. 158-168.

8. Backlund, D.C. and M.M. Gary, *New records of the endangered American burying beetle, Nicrophorus americanus Olivier, (Coleoptera: Silphidae) in South Dakota.* The Coleopterists Bulletin, 1997. **51**(1): p. 53-58.

9. Bedick, J.C., B.C. Ratcliffe, and L.G. Higley, *A new sampling protocol for the endangered American burying beetle, Nicrophorus americanus Olivier (Coleoptera: Silphidae).* The Coleopterists Bulletin, 2004. **58**: p. 57-70.

10. Bedick, J.C., et al., *Distribution, ecology and population dynamics of the American burying beetle [Nicrophorus americanus Olivier (Coleoptera, Silphidae)] in south-central Nebraska, USA.* Journal of Insect Conservation, 1999. **3**: p. 171-181.

11. Keller, M.L., D.R. Howard, and C.L. Hall, *Spatiotemporal niche partitioning in a specious silphid community (Coleoptera: Silphidae Nicrophorus).* The Science of Nature, 2019. **106**(11): p. 57.

12. Jakubec, P. and J. Růžička, *Is the type of soil an important factor determining the local abundance of carrion beetles (Coleoptera: Silphidae)?* European Journal of Entomology, 2015. **112**(4).

13. Kozminykh, V.O. and S.L. Esyunin, *Spectra of ecological groups and the structure of Coleoptera necrobiont communities.* Russian Entomological Journal, 1994. **3**: p. 75-80.

14. Tezcan, S. and J. Hava, *Notes on the pitfall trap collected carrion Beetles (Coleoptera, Silphidae) in ecological cherry orchards in Izmir and Manisa provinces of Turkey.* Ziraat Fakultesi Dergisi, 2001. **38**(1): p. 33-38.

15. Hanski, I. and J. Krikken, *Dung beetles in tropical forests in South-East Asia*, in *Dung Beetle Ecology*, I. Hanski and J. Cambefort, Editors. 1991, Princeton University Press: Princeton, New Jersey, USA. p. 179-197.

16. Bayartogtokh, B. and E. Otgonjargal, *Assemblages of coprophilous beetles (Insecta: Coleoptera) in the pastureland of Central Mongolia.* Mongolian Journal of Biological Sciences, 2009. **7**(1-2): p. 19-27.

17. Arnett, R.H., *Coleoptera notes I: Silphidae.* Canadian Entomologist, 1946. **78**(7-8): p. 131-134.

18. Anderson, R.S. and S.B. Peck, *The carrion beetles of Canada and Alaska. Coleoptera: Silphidae and Agyrtidae*. 1985, Ottawa, ON, Canada: Agriculture Canada.

19. Bishop, A.A., et al., *A comparison of an ecological model and GIS spatial analysis to describe niche partitioning amongst carrion beetles in Nebraska.* Transactions in GIS, 2002. **6**(4): p. 457-470.

20. Lingafelter, S.W., *Diversity, habitat preferences, and seasonality of Kansas carrion beetles (Coleoptera: Silphidae).* Journal of the Kansas Entomological Society, 1995. **68**(2): p. 214-223.

21. Garfinkel, C.F. and C.M. McCain, *Substantial niche overlap in carrion beetle habitat and vegetation use.* Ecological Entomology, 2023. **48**: p. 433-444.

22. Peck, S. and M. Kaulbars, *A synopsis of the distribution and bionomics of the carrion beetles (Coleoptera: Silphidae) of the conterminous United States.* Proceedings of the Entomological Society of Ontario, 1987. **118**: p. 47-81.

23. Peck, S.B. and R.S. Anderson, *Taxonomy, phylogeny and biogeography of the carrion beetles of Latin America (Coleoptera: Silphidae).* Quaestiones entomologicae, 1985. **21**(3): p. 247-318.

24. Su, H., *The morphology, development, behaviors, and interspecific competition of Nicrophorus concolor Kraatz (Coleoptera: Silphidae).* . 2010, National University of Tainan: Tainan, Taiwan.

25. Ito, M., *Frequency of carcass burial in animal burrows for reproduction by Nicrophorus concolor (Coleoptera: Silphidae).* Journal of Ethology, 2021. **39**(1): p. 141-144.

26. Katakura, H., M. Sonoda, and N. Yoshida, *Carrion beetle (Coleoptera, Silphidae) fauna of Hokkaido University Tomakomai experiment forest, Northern Japan, with a note on the habitat preference of a geotrupine species, Geotrupes laevistriatus (Coleoptera, Scarabaeidae).* Hokkaido University Faculty of Agriculture Experimental Forest Research Report, 1986. **43**(1): p. 43-55.

27. Nagano, M. and S. Suzuki, *Phenology and habitat use among Nicrophorine beetles of the genus Nicrophorus and Ptomascopus (Coleoptera: Silphidae).* Edaphologia, 2003. **73**: p. 1-9.

28. Jung, S.-H. and H.-S. Oh, *Insect fauna of Yeongsil in Mt. Hallasan National Park (excluding Lepidoptera).* Journal of Korean Nature, 2012. **5**(1): p. 27-36.

29. Ito, M., *Study of community assembly patterns and interspecific interactions involved in insect succession on rat carcasses.* Entomological Science, 2020. **23**(1): p. 105-116.

30. Scott, M. and J. Traniello, *Behavioural and ecological correlates of male and female parental care and reproductive success in burying beetles (Nicrophorus spp.).* Animal Behaviour, 1990. **39**(2): p. 274-283.

31. Miller, C.J., et al., *Examining transmission of gut bacteria to preserved carcass via anal secretions in Nicrophorus defodiens.* PloS One, 2019. **14**(12): p. e0225711.

32. Trumbo, S.T., *Monogamy to communal breeding: exploitation of a broad resource base by burying beetles (Nicrophorus).* Ecological Entomology, 1992. **17**(3): p. 289-298.

33. Scott, M.P., *The benefit of paternal assistance in intra- and interspecific competition for the burying beetle, Nicrophorus defodiens.* Ethology Ecology & Evolution, 1994. **6**(4): p. 537-543.

34. Wilson, D.S. and J. Fudge, *Burying beetles: intraspecific interactions and reproductive success in the field.* Ecological Entomology, 1984. **9**(2): p. 195-203.

35. Wilson, D.S. and W.G. Knollenberg, *Adaptive indirect effects: the fitness of burying beetles with and without their phoretic mites.* Evolutionary Ecology, 1987. **1**(2): p. 139-159.

36. Lowe, A.J. and R.F. Lauff, *Arboreal burials in Nicrophorus spp. (Coleoptera: Silphidae).* Psyche, 2012. **2012**: p. 1-6.

37. Wilhelm, S.I., D.J. Larson, and A.E. Storey, *Habitat preference of two burying beetles (Coleoptera: Silphidae: Nicrophorus) living among seabirds.* Northeastern Naturalist, 2001. **8**(4): p. 435-442.

38. Wilson, D.S. and W.G. Knollenberg, *Food discrimination and ovarian development in burying beetles (Coleoptera: Silphidae: Nicrophorus).* Annals of the Entomological Society of America, 1984. **77**(2): p. 165-170.

39. Michaud, J.-P., et al., *Natural and anthropogenic changes in the insect fauna associated with carcasses in the North American Maritime lowlands.* Forensic Science International, 2010. **202**(1-3): p. 64-70.

40. Anderson, R.S., *Resource partitioning in the carrion beetle (Coleoptera: Silphidae) fauna of southern Ontario: ecological and evolutionary considerations.* Canadian Journal of Zoology, 1982. **60**(6): p. 1314-1325.

41. Beninger, C.W. and S.B. Peck, *Temporal and spatial patterns of resource use among Nicrophorus carrion beetles (Coleoptera: Silphidae) in a Sphagnum bog and adjacent forest near Ottawa, Canada.* The Canadian Entomologist, 1992. **124**(1): p. 79-86.

42. Wettlaufer, J.D., et al., *Partitioning resources through the seasons: abundance and phenology of carrion beetles (Silphidae) in southeastern Ontario, Canada.* Canadian Journal of Zoology, 2021. **99**(11): p. 961-973.

43. Peck, S.B. and R.S. Anderson, *Taxonomy, phylogeny and biogeography of the carrion beetles of Latin America (Coleoptera: Silphidae)* Quaestiones Entomologicae, 1985. **21**: p. 247-317.

44. Sikes, D.S. and T. Mousseau, *Description of Nicrophorus efferens, new species, from Bougainville Island (Coleoptera, Silphidae, Nicrophorinae).* Zookeys, 2013(311): p. 83-93.

45. Pukowski, E., *Ökologische Untersuchungen an Necrophorus* Zeitschrift für Morphologie und Ökologie der Tiere, 1933. **27**: p. 518-586.

46. Spicarova, N., *Diurnal activity of young individuals of the species Necrophorus germanicus (Col. Silphidae).* Acta Universitatis Palackianae Olomoucensis, 1974. **47**: p. 179-187.

47. Cook, L.M., et al., *Evidence for differential diel activity patterns in two co-occurring species of burying beetles (Coleoptera: Silphidae: Nicrophorinae).* Western North American Naturalist, 2019. **79**(2): p. 270-274.

48. Burke, K.W., A.F. Groulx, and P.R. Martin, *The competitive exclusion–tolerance rule explains habitat partitioning among co‐occurring species of burying beetles.* Ecology, 2024. **105**(1): p. e4208.

49. Collard, A.E., et al., *Body size variation in a guild of carrion beetles.* Canadian Journal of Zoology, 2021. **99**(2): p. 117-129.

50. Burke, K.W., et al., *Habitat use of co-occurring burying beetles (genus Nicrophorus) in southeastern Ontario, Canada.* Canadian Journal of Zoology, 2020. **98**(9): p. 591-602.

51. Sikes, D.S. and S.B. Peck, *Description of Nicrophorus hispaniola, new species, from Hispaniola (Coleoptera: Silphidae) and a key to the species of Nicrophorus of the New World.* Annals of the Entomological Society of America, 2000. **93**(3): p. 391-397.

52. Perez-Gelabert, D.E., *New Hispaniola locality record for the endemic beetle Nicrophorus hispaniola Sikes & Peck, 2000 (Coleoptera: Silphidae: Nicrophorinae).* Novitates Caribaea, 2016(10): p. 92-95.

53. Kocárek, P., *Diurnal activity rhythms and niche differentiation in a carrion beetle assemblage (Coleoptera: Silphidae) in Opava, the Czech Republic.* Biological Rhythm Research, 2001. **32**(4): p. 431-438.

54. Aleksandrowicz, O. and K. Komosinski, *On the fauna of carrion beetles (Coleoptera, Silphidae) of Mazurian lakeland (north-eastern Poland)*, in *Protection of Coleoptera in the Baltic Sea Region*, J. Skłodowski, et al., Editors. 2005, Agricultural University Press: Warsaw. p. 147-153.

55. Ruzicka, J., *Seasonal activity and habitat associations of Silphidae and Leiodidae: Cholevinae (Coleoptera) in central Bohemia.* Acta Societatis Zoologicae Bohemoslovicae, 1994. **58**: p. 67-78.

56. Konieczna, K., Z. Czerniakowski, and P. Wolański, *Assemblages of necrophilous carrion beetles (Col., Silphidae) in agriculturally used areas.* Applied Ecology & Environmental Research, 2019. **17**(2): p. 297-313.

57. Peschke, K. and D. Fuldner, *Ecological separation, functional relationships, and limiting resources in a carrion insect community.* Zoologische Jahrbucher Abteilung fur Systematic Okologie und Geographie der Tiere Jena, 1987. **114**: p. 241-265.

58. Kočárek, P., *Small carrion beetles (Coleoptera: Leiodidae: Cholevinae) in Central European lowland ecosystem: seasonality and habitat preference.* Acta Societatis Zoologicae Bohemicae, 2002. **66**: p. 37-45.

59. Esh, M. and A. Oxbrough, *Macrohabitat associations and phenology of carrion beetles (Coleoptera: Silphidae, Leiodidae: Cholevinae).* Journal of Insect Conservation, 2021. **25**: p. 123-136.

60. Easton, C., *The ecology of burying beetles (Necrophorus: Coleoptera, Silphidae)*. 1979, University of Glasgow.

61. Peck, S.B., *Nicrophorus (Silphidae) can use large carcasses for reproduction (Coleoptera).* The Coleopterists Bulletin, 1986. **40**(1): p. 44-44.

62. Hocking, M., R. Ring, and T. Reimchen, *Burying beetle Nicrophorus investigator reproduction on Pacific salmon carcasses.* Ecological Entomology, 2006. **31**(1): p. 5-12.

63. Smith, R.J. and B. Heese, *Carcass selection in a high altitude population of the burying beetle, Nicrophorus investigator (Silphidae).* The Southwestern Naturalist, 1995. **40**: p. 50-55.

64. Smith, R.J., et al., *Altitudinal variation in body size and population density of Nicrophorus investigator (Coleoptera: Silphidae).* Environmental Entomology, 2000. **29**(2): p. 290-298.

65. Smith, R.J. and M.J. Merrick, *Resource availability and population dynamics of Nicrophorus investigator, an obligate carrion breeder.* Ecological Entomology, 2001. **26**(2): p. 173-180.

66. O'Hanlon, A., et al., *Irish carrion beetles (Coleoptera: Silphidae): A review of the species and their known distribution*, in *Biology and Environment: Proceedings of the Royal Irish Academy*. 2020, Royal Irish Academy. p. 219-235.

67. Katakura, H. and R. Ueno, *A preliminary study on the faunal make-up and spatiotemporal distribution of carrion beetles (Coleoptera: Silphidae) on the Ishikari Coast, Northern Japan.* Japanese Journal of Ecology, 1985. **35**(4): p. 461-468.

68. Katakura, H. and H. Fukuda, *Faunal makeup of ground and carrion beetles in Kamiotoineppu, Hokkaido University Nakagawa Experimental Forest, northern Japan, with some notes on related problems.* Research Bulletins of the College Experimental Forests Hokkaido University, 1975. **32**(1): p. 75-92.

69. Environment, M.o.t., *Threatened Wildlife of Japan. Red Data Book 2014 Insecta.* . 2015, Tokyo: Environment Agency of Japan.

70. Ohkawara, K., S. Suzuki, and H. Katakura, *Competitive interaction and niche differentiation among burying beetles (Silphidae, Nicrophorus) in northern Japan.* Entomological Science, 1998. **1**(4): p. 551-559.

71. Damron, E.P., et al., *No evidence for increased fitness of offspring from multigenerational effects of parental size or natal carcass size in the burying beetle Nicrophorus marginatus.* PLoS One, 2021. **16**(7): p. e0253885.

72. Lomolino, M.V., et al., *Ecology and conservation of the endangered American burying beetle (Nicrophorus americanus).* Conservation Biology, 1995. **9**(3): p. 605-614.

73. Trumbo, S.T. and P.L. Bloch, *Habitat fragmentation and burying beetle abundance and success.* Journal of Insect Conservation, 2000. **4**: p. 245-252.

74. Rintoul, D.A., et al., *Carrion beetles (Coleoptera: Silphidae) of the Konza Prairie Biological Station.* Journal of the Kansas Entomological Society, 2005. **78**(2): p. 124-133.

75. Dyer, N.W. and D.L. Price, *Notes on the diversity and foraging height of carrion beetles (Coleoptera: Silphidae) of the Nassawango Creek Preserve, Maryland, USA.* The Coleopterists Bulletin, 2013. **67**(3): p. 397-400.

76. Bedick, J.C., W.W. Hoback, and M.C. Albrecht, *High water-loss rates and rapid dehydration in the burying beetle, Nicrophorus marginatus.* Physiological Entomology, 2006. **31**(1): p. 23-29.

77. García-Real, E., L.E. Rivera-Cervantes, and C. Palomera-García, *New distributional records for Nicrophorus marginatus Fabricius (Coleoptera: Silphidae) from Jalisco, México.* Entomological News, 2005. **116**(2): p. 107-110.

78. Nishikawa, M. and D.S. Sikes, *New records and range extensions of the Asian Silphidae (Coleoptera).* Special Publication of the Japan Coleopterological Society, Osaka, 2008. **2**: p. 127-143.

79. Anduaga, S., *Reproductive biology of Nicrophorus mexicanus Matthews (Coleoptera: Silphidae).* The Coleopterists Bulletin, 2009. **63**(2): p. 173-178.

80. Halffter, G., S. Anduaga, and C. Huerta, *Nidification des Nicrophorus [Col. Silphidae].* Bulletin de la Société Entomologique de France, 1983. **88**(7): p. 648-666.

81. Palestrini, C., et al., *Nicrophorus mexicanus (Coleoptera: Silphidae: Nicrophorinae): larval morphology and phylogenetic considerations on the N. investigator group.* Acta Societatis Zoologicae Bohemicae, 1996. **60**: p. 435-445.

82. Huerta, C., *Glándulas esternales y comportamiento de nidificación en Nicrophorus mexicanus Matthews (Coleoptera: Silphidae).* Elytron, 1991. **5**: p. 13-21.

83. Anduaga, S. and C. Huerta, *Effect of parental care on the duration of larval development and offspring survival in Nicrophorus mexicanus Matthews (Coleoptera: Silphidae).* The Coleopterists Bulletin, 2001. **55**(3): p. 264-270.

84. Pérez-Villamares, J.C., E. Jiménez-Sánchez, and J. Padilla-Ramírez, *Beetles attracted to carrion (Coleoptera: Scarabaeidae, Geotrupidae, Hybosoridae, Trogidae and Silphidae) in the glens of Coatepec Harinas, Estado de México, Mexico.* Revista Mexicana de Biodiversidad, 2016. **87**(2): p. 443-450.

85. Ren, J., et al., *Beetle data set collected using pitfall trapping in the Gobi Desert of the Hexi Corridor.* Biodiversity Science, 2024. **32**(2): p. 23375.

86. Tsai, H.-Y., et al., *Locally-adapted reproductive photoperiodism determines population vulnerability to climate change in burying beetles.* Nature Communications, 2020. **11**(1): p. 1398.

87. Hwang, W. and S.F. Shiao, *Dormancy and the influence of photoperiod and temperature on sexual maturity in Nicrophorus nepalensis (Coleoptera: Silphidae).* Insect Science, 2011. **18**(2): p. 225-233.

88. Chen, B.F., et al., *A chemically triggered transition from conflict to cooperation in burying beetles.* Ecology Letters, 2020. **23**(3): p. 467-475.

89. Chan, S.-F., et al., *Higher temperature variability in deforested mountain regions impacts the competitive advantage of nocturnal species.* Proceedings of the Royal Society B, 2023. **290**(1999): p. 20230529.

90. Sikes, D.S., *The natural history of Nicrophorus nigrita, a western Nearctic species (Coleoptera: Silphidae).* The Pan Pacific Entomologist, 1996. **72**(2): p. 70-81.

91. Potticary, A.L., et al., *takeout gene expression is associated with temporal kin recognition.* Royal Society Open Science, 2023. **10**(8): p. 230860.

92. Potticary, A.L., C.B. Cunningham, and A.J. Moore, *Offspring overcome poor parenting by being better parents.* Journal of Evolutionary Biology, 2024. **37**(1): p. 100-109.

93. Capodeanu-Nägler, A., et al., *From facultative to obligatory parental care: interspecific variation in offspring dependency on post-hatching care in burying beetles.* Scientific Reports, 2016. **6**(1): p. 29323.

94. Potticary, A.L., et al., *Spatiotemporal variation in the competitive environment, with implications for how climate change may affect a species with parental care.* Ecology and Evolution, 2023. **13**(4): p. e9972.

95. Hoffman, R.L., J.C. Mitchell, and S.C. Kirby, *Relative abundance, habitat preference, and seasonal occurrence of two species of burying beetles in central Virginia (Silphidae).* Banisteria, 2012. **40**: p. 61-65.

96. Shubeck, P.P., *Diel periodicities of certain carrion beetles (Coleoptera: Silphidae).* The Coleopterists' Bulletin, 1971. **25**: p. 41-46.

97. Wettlaufer, J.D., et al., *A test of the competitive ability–cold tolerance trade-off hypothesis in seasonally breeding beetles.* Ecological Entomology, 2023. **48**(1): p. 55-68.

98. Robertson, I.C., *Relative abundance of Nicrophorus pustulatus (Coleoptera: Silphidae) in a burying beetle community, with notes on its reproductive behavior.* Psyche, 1992. **99**(2-3): p. 189-198.

99. Smith, G., et al., *Host shift by the burying beetle, Nicrophorus pustulatus, a parasitoid of snake eggs.* Journal of Evolutionary Biology, 2007. **20**(6): p. 2389-2399.

100. Rauter, C.M. and A.J. Moore, *Quantitative genetics of growth and development time in the burying beetle Nicrophorus pustulatus in the presence and absence of post-hatching parental care.* Evolution, 2002. **56**(1): p. 96-110.

101. Engasser, E.L., R.L. Stone, and M.L. Jameson, *Habitat associations of carrion beetles (Coleoptera: Silphidae) across a full annual cycle.* Environmental Entomology, 2021. **50**(3): p. 605-614.

102. Wormington, J.D. and B. Luttbeg, *Disrupting information alters the behavioral response to a mutual signal trait in both sexes of Nicrophorus (Coleoptera: Silphidae) burying beetles.* Behavioral Ecology, 2019. **30**(4): p. 960-967.

103. Mora-Aguilar, E.F. and L. Delgado, *First state record of Nicrophorus quadrimaculatus Matthews (Coleoptera: Silphidae) from Oaxaca, Mexico.* The Coleopterists Bulletin, 2015. **69**(2): p. 324-324.

104. Takata, M., et al., *A parental volatile pheromone triggers offspring begging in a burying beetle.* iScience, 2019. **19**: p. 1260-1278.

105. Nisimura, T., M. Kon, and H. Numata, *Bimodal life cycle of the burying beetle Nicrophorus quadripunctatus in relation to its summer reproductive diapause.* Ecological Entomology, 2002. **27**(2): p. 220-228.

106. Takata, M., et al., *The proximate cause of asynchronous hatching in the burying beetle Nicrophorus quadripunctatus.* Journal of Ethology, 2015. **33**(3): p. 197-203.

107. Suzuki, S., *Changing dominant–subordinate relationships during carcass preparation between burying beetle species (Nicrophorus: Silphidae: Coleoptera).* Journal of Ethology, 2000. **18**(1): p. 25-28.

108. Nisimura, T., H. Numata, and E. Yoshioka, *Effect of temperature on circadian rhythm controlling the crepuscular activity of the burying beetle Nicrophorus quadripunctatus Kraatz (Coleoptera: Silphidae).* Entomological Science, 2005. **8**(4): p. 331-338.

109. Benowitz, K.M., et al., *Development and the effects of extended parenting in the cold-breeding burying beetle Nicrophorus sayi.* Ecological Entomology, 2019. **44**(1): p. 11-16.

110. Scott, M.P., *Competition with flies promotes communal breeding in the burying beetle, Nicrophorus tomentosus.* Behavioral Ecology and Sociobiology, 1994. **34**: p. 367-373.

111. Oldekop, J.A., et al., *Adaptive switch from infanticide to parental care: how do beetles time their behaviour?* Journal of Evolutionary Biology, 2007. **20**(5): p. 1998-2004.

112. Smiseth, P.T., R.J.S. Ward, and A.J. Moore, *Asynchronous hatching in Nicrophorus vespilloides, an insect in which parents provide food for their offspring.* Functional Ecology, 2006. **20**(1): p. 151-156.

113. Lock, J.E., P.T. Smiseth, and A.J. Moore, *Selection, inheritance, and the evolution of parent-offspring interactions.* The American Naturalist, 2004. **164**(1): p. 13-24.

114. Parker, D.J., et al., *Transcriptomes of parents identify parenting strategies and sexual conflict in a subsocial beetle.* Nature Communications, 2015. **6**(1): p. 8449.

115. Eggert, A.-K., M. Reinking, and J.K. Müller, *Parental care improves offspring survival and growth in burying beetles.* Animal Behaviour, 1998. **55**(1): p. 97-107.

116. Sikes, D., S. Trumbo, and S. Peck, *Cryptic diversity in the New World burying beetle fauna: Nicrophorus hebes Kirby-new status as a resurrected name (Coleoptera: Silphidae: Nicrophorinae).* Arthropod Systematics & Phylogeny, 2016. **74**: p. 299-309.

117. Peck, S.B., *The life history of the Japanese carrion beetle Ptomascopus morio and the origins of parental care in Nicrophorus (Coleoptera, Silphidae, Nicrophorini).* Psyche, 1982. **89**(1-2): p. 107-111.

118. Trumbo, S.T., M. Kon, and D. Sikes, *The reproductive biology of Ptomascopus morio, a brood parasite of Nicrophorus.* Journal of Zoology, 2001. **255**(4): p. 543-560.
